# Allelic Variation in Developmental Genes and Effects on Winter Wheat Heading Date in the U.S. Great Plains

**DOI:** 10.1371/journal.pone.0152852

**Published:** 2016-04-08

**Authors:** Sarah M. Grogan, Gina Brown-Guedira, Scott D. Haley, Gregory S. McMaster, Scott D. Reid, Jared Smith, Patrick F. Byrne

**Affiliations:** 1 Soil and Crop Sciences, Colorado State University, Fort Collins, Colorado, United States of America; 2 Regional Small Grains Genotyping Laboratory, USDA-ARS, Raleigh, North Carolina, United States of America; 3 Agricultural Systems Research Unit, USDA-ARS, Fort Collins, Colorado, United States of America; Institute of Genetics and Developmental Biology, CHINA

## Abstract

Heading date in wheat (*Triticum aestivum* L.) and other small grain cereals is affected by the vernalization and photoperiod pathways. The reduced-height loci also have an effect on growth and development. Heading date, which occurs just prior to anthesis, was evaluated in a population of 299 hard winter wheat entries representative of the U.S. Great Plains region, grown in nine environments during 2011–2012 and 2012–2013. The germplasm was evaluated for candidate genes at vernalization (*Vrn-A1*, *Vrn-B1*, and *Vrn-D1*), photoperiod (*Ppd-A1*, *Ppd-B1* and *Ppd-D1*), and reduced-height (*Rht-B1* and *Rht-D1*) loci using polymerase chain reaction (PCR) and Kompetitive Allele Specific PCR (KASP) assays. Our objectives were to determine allelic variants known to affect flowering time, assess the effect of allelic variants on heading date, and investigate changes in the geographic and temporal distribution of alleles and haplotypes. Our analyses enhanced understanding of the roles developmental genes have on the timing of heading date in wheat under varying environmental conditions, which could be used by breeding programs to improve breeding strategies under current and future climate scenarios. The significant main effects and two-way interactions between the candidate genes explained an average of 44% of variability in heading date at each environment. Among the loci we evaluated, most of the variation in heading date was explained by *Ppd-D1*, *Ppd-B1*, and their interaction. The prevalence of the photoperiod sensitive alleles *Ppd-A1b*, *Ppd-B1b*, and *Ppd-D1b* has gradually decreased in U.S. Great Plains germplasm over the past century. There is also geographic variation for photoperiod sensitive and reduced-height alleles, with germplasm from breeding programs in the northern Great Plains having greater incidences of the photoperiod sensitive alleles and lower incidence of the semi-dwarf alleles than germplasm from breeding programs in the central or southern plains.

## Introduction

Hexaploid wheat is a widely cultivated, productive, and nutritionally important crop grown across most major agricultural regions of the world. In 2013 wheat was planted on more than 290 million hectares worldwide, which is more land than any other crop (http://faostat3.fao.org). Average global yield was estimated at 3.27 t ha^-1^. In the U.S. average yield was 3.17 t ha^-1^ for all classes of wheat and 2.46 t ha^-1^ for hard red winter wheat (http://www.ers.usda.gov), the predominant market class in the Great Plains region. The global success of wheat can be attributed to its adaptability to diverse management practices and environmental conditions [1].

Variation in flowering time tailors wheat to a particular target environment. Floral organs are susceptible to environmental stresses, and freezing temperatures, drought stress, or heat stress can damage delicate floral structures and reduce yield. Local adaptation can arise from direct or indirect selection of developmental timing best suited for local temperature and precipitation patterns, management practices, soil properties, or other environmental characteristics [2]. Fine-tuning plant development may increase yield and yield stability by timing growth and development around the type, onset, duration, and severity of stresses characteristic of the region. Wheat breeders typically select (directly or indirectly) for later flowering in northern regions to protect the developing spike from cold temperatures and earlier development at southern latitudes where it is more important to flower before the onset of heat stress. Later flowering is also associated with a delay in timing of other developmental events, such as the initiation of spikelet and floret primordia at double ridge, or other phenological events such as jointing, booting, and heading [3]. Later flowering can also affect yield potential by modifying spike and spikelet formation, such as by affecting the total number [4] or arrangement [5] of spikelet primordia initiated, or the number of fertile florets produced [6]

Three major genetic systems affect flowering time in wheat: vernalization, photoperiod, and earliness *per se* (EPS). Vernalization requirements control winter, spring, or facultative growth habit and are governed by at least three groups of loci (*Vrn1*, *Vrn2*, and *Vrn3*) [7]. Ancestral wheats were sensitive to vernalization and required a period of continuous cold temperature to transition from vegetative to reproductive growth. The most common source of spring growth habit (i.e., not requiring vernalization) is a dominant mutation at one or more *Vrn1* loci (*Vrn-A1*, *Vrn-B1*, *Vrn-D1*), located on the long arm of the group 5 chromosomes [8–9]. Variation at other vernalization loci also contribute to quantitative variation in flowering time [10]. Dominant mutations at *Vrn3* loci [11] reduce the vernalization requirement and recessive mutations at *Vrn2* loci [12–13] accelerate flowering time.

The photoperiod sensitivity system also provides broad adaptability to specific environmental conditions. Three homoeologous photoperiod loci (*Ppd-A1*, *Ppd-B1*, *Ppd-D1*) are located on the group 2 chromosomes [14]. Ancestral wheats were photoperiod sensitive and required long days to flower. Mutations at the photoperiod loci cause the photoperiod insensitive “day neutral” phenotype associated with earlier flowering in some modern-day bread wheats. Variation in photoperiod sensitivity is primarily controlled by allelic variants at *Ppd-D1* [15] and copy number variants at *Ppd-B1* [16]. Allelic variation at *Ppd-A1* also affects photoperiod sensitivity, however the allelic effects of *Ppd-A1* are weaker than *Ppd-D1* or *Ppd-B1* [17]. The photoperiod and vernalization pathways are integrated and epistatic interactions between photoperiod loci and *Vrn1* loci are well characterized [18].

Additional variation in flowering time after vernalization and photoperiod requirements have been fulfilled is due to EPS. These small effect loci are numerous and heritable [2], but generally unstable across environments [19]. Less is known about EPS than the other genetic systems affecting flowering time. Further optimization of developmental timing is contributed by epistatic interactions among genes or QTL [19] and may be affected by gibberellin insensitivity through the reduced-height loci [20].

The reduced-height loci are known to affect growth and development in winter wheat [21]. Reduced-height genes encode DELLA proteins, which are growth repressors that are degraded by a process that involves gibberellin [22]. The reduced-height alleles *Rht-B1b* and *Rht-D1b* each have a single SNP that causes a premature stop codon, resulting in reduced sensitivity to gibberellic acid and therefore, semi-dwarf stature. Gibberellin insensitivity has been associated with earlier heading date in wheat, and *Rht-B1b* and *Rht-D1b* are the predominant semi-dwarf alleles in wheat worldwide [20]. *Rht-B1b* has been reported as the leading cause of semi-dwarf stature among winter wheat in the U.S. Great Plains region [23]. Guedira et al. [23] found the reduced-height alleles *Rht-B1b* and *Rht-D1b* to be widespread in U.S. germplasm, and *Rht8c* to be less common but linked with the photoperiod insensitive allele *Ppd-D1a*.

The allelic diversity of candidate loci known to affect heading date has been characterized in several worldwide [20, 24] and regional [25–27] collections of spring and winter wheat. Analyses of core collections of germplasm have indicated substantial variation in the geographic distribution of vernalization and photoperiod alleles. Variation in the presence and distribution of alleles and haplotypes has been found to vary among continents e.g.,[24] and countries [26], with some haplotypes under-represented or absent from particular geographic regions. A more complete understanding of the genetic controls—including the allelic variants and effects of single genes, and distribution of favorable multi-locus genotypes—is important for plant breeders to prepare for future climate scenarios including those that are more variable or extreme than today’s conditions [28–29].

We examined allelic variation, distribution, and effects of vernalization, photoperiod, and reduced-height loci in a collection of hard winter wheat germplasm representative of the U.S. Great Plains. This is the first article to determine allelic diversity at the photoperiod loci in winter wheat germplasm from the U.S. Great Plains. The objectives of the experiment were

1. To characterize allelic variants at loci known to affect flowering time, including vernalization, photoperiod, and reduced-height genes;
2. To assess the effect of allelic variants on heading dates in nine U.S. Great Plains environments; and
3. To investigate temporal and geographic distribution of alleles and haplotypes among germplasm developed during different periods or from different regions of the U.S. where hard winter wheat is grown.

## Materials and Methods

### Germplasm

The germplasm was a collection of 299 winter wheat genotypes (or “entries”) comprising the Triticeae Coordinated Agricultural Project (http://www.triticeaecap.org) hard winter wheat association mapping panel (S1 Table). The entries included modern and historic cultivars and experimental breeding lines. Public breeding programs contributed 270 entries, private breeding programs contributed 27, and two were historic cultivars developed before 1900. The publically developed germplasm came from Nebraska (55 entries), Oklahoma (54 entries), Texas (51 entries), Colorado (34 entries), Kansas (30 entries), Montana (23 entries), South Dakota (21 entries), Michigan (1 entry), and North Dakota (1 entry). The entries were derived between 1874 and 2010, but most represent modern improvements and were derived after the Green Revolution. Only 19 were derived before 1960.

### Environments and Experimental Design

The entries were evaluated in nine field trials conducted across the U.S. Great Plains region during 2011–2012 and 2012–2013, as described previously [30] and in Table 1**.** Each environment was a combination of location, year, and moisture treatment, with three possible moisture regimes: full irrigation, partial irrigation, and rainfed (no supplemental irrigation). Environments grown in 2011–2012 include rainfed in Bushland, TX (Bu12R), full irrigation in Greeley, CO (Gr12F), partial irrigation in Greeley, CO (Gr12P), rainfed in Ithaca, NE (It12R), and Manhattan, KS (Ma12). The 2012–2013 environments include rainfed in Ardmore, OK (Ar13R), Fort Collins, CO (Fo13), rainfed in Hays, KS (Ha13R), and rainfed in Ithaca, NE (It13R). Different environments at the same location during the same year were side-by-side treatments with a moisture differential. There were separate rainfed and irrigated treatments at Fo13 and Ma12, but irrigation was not applied until after heading date so there were no significant treatment effects on heading date and the moisture treatments were treated as replications.

**Table 1:** Description of environments where field trials were grown. Environment abbreviation, location, moisture treatment, latitude and longitude, and planting and harvest dates.

Environment	Location	Moisture Treatment	Lat (°N)	Long (°W)	Planting Date	Harvest Date
Ar13R	Ardmore, OK	Rainfed	34.18	-97.09	12 Oct 2012	25 June 2013
Bu12R	Bushland, TX	Rainfed	35.18	-102.10	3 Nov 2011	10 June 2012
Fo13	Fort Collins, CO	Averaged across treatments^1^	40.65	-105.00	2 Oct 2012	18–22 July 2013^2^
Gr12P	Greeley, CO	Partial irrigation	40.42	-104.71	19 Oct 2011	3 July 2012
Gr12F	Greeley, CO	Full irrigation	40.42	-104.71	19 Oct 2011	13 July 2012
Ha13R	Hays, KS	Rainfed	38.88	-99.33	10 Oct 2012	3 July 2013
It12R	Ithaca, NE	Rainfed	41.16	-96.43	4 Oct 2011	28 June 2012
It13R	Ithaca, NE	Rainfed	41.28	-96.41	25 Sept 2012	17 July 2013
Ma12	Manhattan, KS	Averaged across treatments	39.14	-96.64	18 Nov 2011	3 July 2012

^1 ‘^Averaged across treatments’ indicates that separate side-by-side rainfed and full-irrigation treatments at this location did not differ significantly for average heading date, so were treated as two replications.

^2^ Rainfed treatment was harvested on 18 July 2013 and fully irrigated treatment was harvested on 22 July 2013.

Four environments (Bu12R, Gr12P, Gr12F, Ha13R) were unreplicated and arranged in an augmented row–column design with two check varieties. The experimental entries were unreplicated except for ‘Wichita’ (CI 11952), which was included in the panel twice. The two check varieties were replicated 15 times each and systematically placed throughout the field. Fo13 had a similar experimental design but included two replications. The check varieties at Bu12R, Fo13, Gr12P, Gr12F, and Ha13R were ‘Hatcher’ [31] and ‘Settler CL’ [32], and these varieties were also included as experimental entries in the trial. Irrigation was applied at Gr12P and Gr12F using drip irrigation. Gr12P was irrigated less frequently and at a reduced volume than Gr12F. Irrigation totaled 101.6 mm at Gr12P and 335.3 mm at Gr12F. It12R and It13R used a similar experimental design but plots were arranged as 15 incomplete blocks, with one plot within each block planted to each of the check varieties Settler CL and ‘Jagger’ [33]. Both It12R and It13R included four replications, and these trials are described in detail by Guttieri et al. [34]. Ma12 had four replications of a modified row-column design arranged as incomplete blocks using a single, locally adapted check variety, ‘Everest’ (http://kswheatalliance.org/varieties/everest/). The Ar13R trial was arranged as a randomized complete block design with two replications. Harvested plot area ranged from 2.2 to 4.6 m^2^.

### Phenotypic Evaluation

Crop development was determined for each field plot using Zadoks’ scale [35]. Heading date (stage 59) was recorded when the spike had fully emerged from the flag leaf sheath in approximately 50% of tillers. Days to heading were recorded as the number of days from January 1 to heading date. Growing degree-days from 1 January to heading were determined previously, and found to be strongly correlated with days from January 1 to heading [30]. However, number of days to heading was used in these analyses because estimates of allelic effects in days are more widely understood than estimates in °C days.

### Genetic Evaluation

Genotypes of nine loci associated with flowering time were obtained primarily from KASP analysis, as described below. Those results were supplemented with polymerase chain reaction (PCR)-based analysis from a previous published report [21] and unpublished data from the Byrne lab at Colorado State University and the USDA-ARS Small Grains Genotyping lab in Manhattan, KS.

#### DNA preparation for genetic analyses

Genomic DNA was extracted at Colorado State University from leaf tissue from single seedlings using the phenol–chloroform method modified slightly from Riede and Anderson [36].

#### KASP marker analysis

Polymorphisms were identified using LGC Genomics (http://www.lgcgroup.com) KASP system fluorescent assays at the USDA-ARS Regional Small Grains Genotyping Laboratory in Raleigh, NC. PCR was run according to manufacturer’s instructions, using a reaction volume of 4.0 μL, which consisted of 2 μL 2x KASPar reaction mix, 0.05 μL 72x assay mix, and 2 μL of template DNA (10 ng μL^-1^). Endpoint genotyping was conducted from fluorescence using the software KlusterCaller (LGC Genomics, Hoddeson, UK).

Kompetitive Allele Specific PCR (KASP) assays developed from published sequences of causal genes were run to distinguish alleles at *Vrn-A1*, *Vrn-B1*, *Vrn-D1*, *Ppd-A1*, *Ppd-B1*, *Ppd-D1*, *Rht-B1*, and *Rht-D1* (Table 2, S2 Table). The exception was the KASP assay wMAS000033 used for detection of the *Vrn-A1a* spring allele developed from the contextual sequences of iSelect SNP marker IWA0001 [37] determined to be associated with *Vrn-A1a*.

**Table 2:** Description of photoperiod (*Ppd)*, reduced-height (*Rht)*, and vernalization (*Vrn)* loci, alleles, and phenotypes evaluated in this study.

Locus	Allele	Phenotype	Reference
*Ppd-A1*	*Ppd-A1a*	photoperiod insensitive	[38–39]
	*Ppd-A1a*.*1*	photoperiod insensitive	[40]
	*Ppd-A1b*	photoperiod sensitive	[39]
*Ppd-B1*	*Ppd-B1a*	photoperiod insensitive	Welsh 1973 [15]
	*Ppd-B1b*	photoperiod sensitive	Welsh 1973 [15]
*Ppd-D1*	*Ppd-D1a*	photoperiod insensitive	Welsh 1973 [15, 38]
	*Ppd-D1b*	photoperiod sensitive	Welsh 1973 [15, 38]
*Rht-B1*	*Rht-B1a*	tall	[41,42]
	*Rht-B1b*	semi-dwarf	[41,42]
*Rht-D1*	*Rht-D1a*	tall	[41,42]
	*Rht-D1b*	semi-dwarf	[41,42]
*Vrn-A1*	*Vrn-A1*	spring growth habit	[43,44]
	*vrn-A1*	winter growth habit	[43]
	*Vrn-A1a*	spring growth habit	[9]
	*Vrn-A1b*	spring growth habit	[9]
	*vrn-A1w*	winter growth habit with higher freezing tolerance, Wichita-type	[45, 46]
	*vrn-A1v*	winter growth habit, Veery- or Jagger-type	[45,46]
	*vrn-A1*, CNV = 1	winter growth habit with earlier flowering	[16, 43]
	*vrn-A1*, CNV = 2	winter growth habit with later flowering	[16, 43]
	*vrn-A1*, CNV>2	winter growth habit with later flowering	[16, 43]
*Vrn-B1*	*Vrn-B1a*	spring growth habit	[47]
	*Vrn-B1b*	spring growth habit	[47]
	*Vrn-B1c*	spring growth habit	[48]
	*vrn-B1-Neuse*	winter growth habit, Neuse-type	[49]
	*vrn-B1-AGS2000*	winter growth habit, AGS2000-type	[49]
*Vrn-D1*	*Vrn-D1*	spring growth habit	[43]
	*vrn-D1*	winter growth habit	[43]

The spring allele *Vrn-A1b* was distinguished from *Vrn-A1a* and the *vrn-A1* winter allele using codominant marker wMAS000034 [4]. Two additional markers were evaluated to determine copy number variation (CNV) of the winter allele *vrn-A1*: Vrn-A1_Exon4_C/T and Vrn-A1_Exon7_G/A [9]. The C allele at *Vrn-A1_exon4* is associated with two or fewer copies, and the G allele at *Vrn-A1_exon7* is associated with a single copy, so entries were classified as having three or more copies, two copies, or one copy of *vrn-A1*. A PCR assay for *Vrn-A1* [45] was also run that detected the same SNP polymorphism and distinguished two winter alleles—renamed *vrn-A1w* and *vrn-A1v* by Eagles et al. [46]—associated with winter dormancy release and freezing tolerance. Thus, the winter alleles detected *at Vrn-A1* using PCR correspond with CNV detected using KASP.

Three KASP markers used to distinguish spring alleles at *Vrn-B1*. TaVrn-B1_D-I, wMAS000037, and Vrn-B1_C are codominant markers that detect the *Vrn-B1a*, *Vrn-B1b* [47], and *Vrn-B1c* [48] spring alleles, respectively. An additional codominant marker, TaVrn-B1_1752, detected an A/G polymorphism in intron 1 of the *vrn-B1* gene associated with differences in vernalization requirement duration [49]. The spring and winter alleles at *Vrn-D1* were distinguished using a single dominant marker, wMAS000039 [43].

Photoperiod insensitive allele *Ppd-A1a*.*1* was assayed with the marker TaPpd-A1prodel, which detects a deletion characteristic of the insensitive allele [50]. Alleles at *Ppd-B1* were distinguished using two markers: wMAS000027, which detects the ‘Chinese Spring’-type insensitive allele with a truncated copy and TaPpdBJ003, which identifies the ‘Sonora 64’-type insensitive allele based on the presence of an intercopy junction [16]. Photoperiod sensitive and insensitive alleles at *Ppd-D1* were distinguished using a single codominant marker, wMAS000024 that detects a deletion upstream of the coding region responsible for the photoperiod insensitive phenotype [15].

Single markers were also used to detect point mutations at the reduced-height loci *Rht-B1* and *Rht-D1* [51]. Mutants at *Rht-B1* were genotyped using wMAS000001 that detected the causative SNP for semi-dwarf stature. Likewise, a single marker, wMAS000002 was used to detect the polymorphism at *Rht-D1* that is associated with semi-dwarf stature. Additional information about KASP assays having wMAS designations is available at http://www.cerealsdb.uk.net/cerealgenomics/CerealsDB/kasp_download.php.

### Diagnostic Markers for Candidate Genes

PCR assays for *Ppd-D1*, *Rht-B1*, *Rht-D1*, and *Vrn-A1* were performed at Colorado State University and complemented results from the KASP assays by confirming allele calls, filling in missing data, and detecting alternate alleles. PCR assays for *Ppd-D1* were conducted as described previously [15] to differentiate the photoperiod insensitive *Ppd-D1a* and two sensitive alleles based on band size of the PCR product. The major photoperiod sensitive allele is *Ppd-D1b*. Detection of the reduced-height alleles *Rht-B1b* and *Rht-D1b* followed the methods of Ellis et al. [51].

### Statistical Analyses

Best linear unbiased predictors were calculated separately for each environment based on field design and spatial trends using SAS 9.3 (SAS Institute, Inc., Cary, NC). Six different spatial correlation models (row-column, spherical, exponential, power, anisotropic power, and Matérn, [52] were tested for environments with an augmented row–column design (Bu12R, Fo13, Gr12P, Gr12F, Ha13). The best model was selected based on the AIC fit statistic. Replications were treated as a random effect for environments with multiple replications (Ar13R, Fo13, It12R, It13R, Ma12).

Further statistical analyses were performed using the R version 3.1.3 [53]. Combined analyses of the effects of alleles at single genes were evaluated on all entries with homozygous allele calls using the ‘car’ package [54]. The ANOVA model terms consisted of the environment, gene, and when significant, interaction between the gene and environment. All terms were fit as fixed effects. Entries with heterozygous calls were treated as missing. When a gene had more than two alleles, pairwise comparisons were run using the ‘lsmeans’ package [55] to test differences between each pair of alleles. Individual environments were analyzed using the linear model (lm) function in the ‘stats’ package [53] to evaluate the proportion of variability in heading date explained by one or more gene, and to test for interactions between genes.

Little’s missing completely at random test (MCAR) was conducted using the ‘BaylorEdPsych’ package [56] to ensure that genotypic data were missing at random. The MCAR test was evaluated on all entries with at least one genotyped allele (297 entries total) and all loci with varying allele calls (*Ppd-A1*, *Ppd-B1*, *Ppd-D1*, *Rht-B1*, *Rht-D1*, *vrn-A1 (*winter alleles and CNV), and *vrn-B1* (winter alleles)); the data were found to be missing at random (Χ^2^ = 5.40, *P =* 1.00*)*. The MCAR test was also evaluated on a subset of 293 entries with one or more genotype at *Ppd-A1*, *Ppd-B1*, *Ppd-D1*, *Rht-B1*, or *Rht-D1*, which were also found to be missing data at random (Χ^2^ = 3.98, *P =* 0.91*)*. Missing genotypic data are believed to be from poor quality or low quantity of DNA for marker assays, and not due to variation in heading date.

Models that tested effects of multiple genes included all entries with complete genotypic data at those loci. For model comparison a subset of 280 entries with complete genotypic data and all homozygous calls at the photoperiod and reduced-height loci were analyzed (Table 3). First, a full model that included the main effects of all five genes and all ten two-way interactions were fit to each data set. In the combined analysis, the main effect of the environment, two-way interactions between the environment and each gene, and ten three-way interactions between the environment and pairs of genes were also included in the model. Then, model selection was applied in R using the ‘MuMIn’ package [57] using the ‘dredge’ function, and the best-fit model was identified based on lowest AIC value. In some cases the best-fit model included non-significant terms, but removing these terms detracted from model fit. The relative importance of main effects in the best-fit model was determined through model averaging, using Akaike weights weighted across all evaluated models. The percent variation accounted for by each model term was calculated as the percent of sums of squares for a given term relative to the total sums of squares or to the sums of squares for all genetic terms in the model.

**Table 3:** Description of multi-locus genotypes at photoperiod and reduced-height loci. The photoperiod (*Ppd*) alleles are ‘*a*’ insensitive and ‘*b*’ sensitive. The reduced-height (*Rht*) alleles are ‘*a*’ tall wild type and ‘*b*’ semi-dwarf. ‘*Het*’ is heterozygous at the locus. Of 299 total entries, 285 entries have complete genotypic data at all five loci, and 280 have complete data with all homozygous allele calls.

n	*Ppd-A1*	*Ppd-B1*	*Ppd-D1*	*Rht-B1*	*Rht-D1*
40	*b*	*b*	*b*	*a*	*a*
4	*b*	*b*	*b*	*a*	*b*
58	*b*	*b*	*b*	*b*	*a*
1	*b*	*b*	*b*	*het*	*a*
6	*b*	*b*	*a*	*a*	*a*
6	*b*	*b*	*a*	*a*	*b*
43	*b*	*b*	*a*	*b*	*a*
1	*b*	*b*	*a*	*het*	*a*
1	*b*	*b*	*a*	*het*	*het*
15	*b*	*a*	*b*	*a*	*a*
1	*b*	*a*	*b*	*a*	*b*
75	*b*	*a*	*b*	*b*	*a*
2	*b*	*a*	*a*	*a*	*a*
2	*b*	*a*	*a*	*a*	*b*
24	*b*	*a*	*a*	*b*	*a*
1	*a*	*b*	*b*	*a*	*het*
1	*a*	*b*	*a*	*a*	*b*
3	*a*	*a*	*b*	*b*	*a*
1	*a*	*a*	*b*	*het*	*a*

## Results

### Allelic Diversity of Candidate Genes

The 299 winter wheat entries were genotyped at 11 candidate genes, but sample size varied among the loci due to different amounts of missing data at each gene. Genotypic data were missing from up to 14 entries per locus. The genotypes at each candidate gene for every entry are provided in S1 Table.

Winter growth habit is determined by recessive alleles at all three *Vrn1* genes. Spring alleles at *Vrn-A1*, *Vrn-B1*, or *Vrn-D1* were not detected for any entry, validating our assumption that this germplasm consists exclusively of winter wheat entries. There was variation among winter alleles at *vrn-A1* and *vrn-B1*. The SNP in *vrn-A1* associated with copy number [9] varied, with 264 (89%) of entries predicted to have three or more copies, four entries (1%) having two copies, and 29 entries (10%) having a single copy. Copy number variation corresponds with the winter alleles at *Vrn-A1* described by Eagles et al. [46], such that the strong winter Wichita allele (*Vrn-A1w*) is associated with three or more copies of the gene and the ‘Veery’ [58] allele (*Vrn-A1v*) with two or fewer copies. Increased copy number at *vrn-A1* has been associated with greater vernalization requirements, resulting in later flowering when the vernalization requirement is only partially fulfilled [16].

Two winter alleles at *Vrn-B1* were previously described to affect heading date following a short vernalization duration, but with no effect when the vernalization requirement is fully satisfied [49]. The winter allele characteristic of ‘AGS2000’ [59] has an A at position 1752 and was associated with lower vernalization requirements and earlier heading than the ‘NC-Neuse’ [60] allele (C at position 1752). Low variation at this locus was observed in this germplasm. Most of the germplasm (291 entries, 99%) carried *vrn-B1-Neuse*; only ‘TAM 401’ [61] and TX05A001822 had *vrn-B1-AGS2000*.

Alleles at the photoperiod genes *Ppd-A1*, *Ppd-B1*, and *Ppd-D1* were polymorphic among the wheat entries evaluated. A total of 291 entries were genotyped successfully at *Ppd-A1*, including 285 entries (98%) with the sensitive allele *Ppd-A1b* and six entries (2%) with the insensitive allele *Ppd-A1a*. The six entries all originated from breeding programs in the southern U.S. and included ‘TAM 302’ [62], OK05303, OK05134, TX04M10211, TX05V7269, and TX06A001132. For *Ppd-B1*, 285 entries were genotyped, including 162 entries (57%) with the sensitive allele *Ppd-B1b* and 123 entries (43%) with the insensitive allele *Ppd-B1a*. Genotypes were obtained at *Ppd-D1* for all 299 entries. Most entries (211 entries, 71%) carried the photoperiod sensitive allele *Ppd-D1b*, while 88 entries (29%) carried *Ppd-D1a*.

The KASP assay identified the photoperiod insensitive allele at *Ppd-D1*. The PCR assay for *Ppd-D1* further characterized allelic variation. Photoperiod insensitivity at *Ppd-D1* is caused by a 2089 bp deletion upstream of the coding region [15]. In presence of the deletion, a 288 bp band is produced that corresponds with *Ppd-D1a*. The presence of the photoperiod sensitive *Ppd-D1b* allele is detected by amplifying a 414 bp fragment within the deletion region with primer pairs Ppd-D1_F and Ppd-D1_R1. *Ppd-D1a* was identified in 88 entries (29%), and the sensitive allele *Ppd-D1b* was found in 204 entries (68%). Guo et al. [40] described two small (24 and 15 bp) insertions within the intact 2089 bp region characteristic of an alternate photoperiod sensitive allele that results in a PCR fragment size of 453 bp. The alternate photoperiod insensitive allele was detected in seven entries (2%).

Both *Rht-B1* and *Rht-D1* were polymorphic among the germplasm evaluated. A total of 294 entries were genotyped for *Rht-B1*, and 296 entries were successfully genotyped for *Rht-D1*. For *Rht-B1*, 211 entries (72%) carried homozygous copies of the semi-dwarf allele *Rht-B1b*, 79 entries (27%) carried homozygous copies of *Rht-B1a*, and four entries (1%) were heterozygous. For *Rht-D1*, 280 entries (95%) carried homozygous copies of the tall wild type allele *Rht-D1a*, 14 entries (5%) carried homozygous copies of the semi-dwarf allele *Rht-D1b*, and two entries (<1%) were heterozygous.

### Diversity of Multi-Locus Genotypes

There were 285 entries (280 entries with all homozygous allele calls and five entries with heterozygous allele calls at one or more loci) with complete genotypic data at all five photoperiod and reduced-height loci. These 285 entries were used to investigate diversity of multi-locus genotypes. Fourteen of the 32 possible combinations of five-locus genotypes with all homozygous calls were present in the germplasm (Table 3). Five additional genotypes had heterozygous allele calls at *Rht-B1* or *Rht-D1*. The most common genotypes were *Ppd-A1b/Ppd-B1a/Ppd-D1b/Rht-B1b/Rht-D1a* (75 entries, 26%), *Ppd-A1b/Ppd-B1b/Ppd-D1b/Rht-B1b/Rht-D1a* (58 entries, 20%), *Ppd-A1b/Ppd-B1b/Ppd-D1a/Rht-B1b/Rht-D1a* (43 entries, 15%), and *Ppd-A1b/Ppd-B1b/Ppd-D1b/Rht-B1a/Rht-D1a* (40 entries, 14%). All other five-locus genotypes had six or fewer entries each.

The most common three-locus genotype at the photoperiod loci was *Ppd-A1b/Ppd-B1b/Ppd-D1b* (103 entries, 36%, Table 3). There were 91 entries (32%) with *Ppd-A1b/Ppd-B1a/Ppd-D1b* and 57 entries (20%) with *Ppd-A1b/Ppd-B1b/Ppd-D1a*. There were 28 entries (10%) with *Ppd-A1b/Ppd-B1a/Ppd-D1a*. Only six entries (2.0%) carried *Ppd-A1a* (with or without insensitive alleles at any other photoperiod gene). No entries had insensitive alleles at all three photoperiod genes.

Among the 285 entries with complete allelic data at all five photoperiod and reduced-height loci, the most common allelic combination at the two reduced-height genes was *Rht-B1b/Rht-D1a* (203 entries, 71%), followed by *Rht-B1a/Rht-D1a* (63 entries, 22%), and *Rht-B1a/Rht-D1b* (14 entries, 5%, Table 3). The remaining entries were heterozygous at one or both reduced-height genes. No entries had mutations at both *Rht-B1* and *Rht-D1*. These results are in agreement with those presented by Guedira et al. [23] in a similar set of germplasm.

### Changes in Diversity of Photoperiod Alleles Over Time

The prevalence of the photoperiod sensitive alleles *Ppd-A1b*, *Ppd-B1b*, and *Ppd-D1b* has decreased gradually among U.S. Great Plains hard winter wheat germplasm over the past century (Table 4). The first appearance of *Ppd-A1a* in this germplasm collection occurred with the derivation of ‘TAM 301’ [63] in 1991 (S1 Table). *Ppd-A1a* is rare in this germplasm but the prevalence of this allele has increased over time. Among entries derived after 1999, 4% were found to carry *Ppd-A1a* (Table 4). The photoperiod sensitive allele *Ppd-B1b* is found in 89% of entries derived prior to 1960, 67% of entries derived between 1960 and 1979, and 58% of those derived between 1980 and 1999. Among germplasm derived after 1999, *Ppd-B1a* is the more common allele and is found in 52% of entries. The prevalence of *Ppd-D1b* is similar among the 19 entries derived before 1960 (84%) as the 33 entries derived between 1960 and 1979 (85%). However, the percentage of entries carrying *Ppd-D1b* drops to 70% among entries derived between 1980 and 1999, and is further reduced to 64% among entries derived in or after 2000.

**Table 4:** Number of entries (n) derived during four different time periods, and the proportion of entries in each group with the photoperiod sensitive allele at *Ppd-A1*, *Ppd-B1*, or *Ppd-D1*. All 285 entries had complete genotypic data at all three loci.

Derivation Period	n	*Ppd-A1b*	*Ppd-B1b*	*Ppd-D1b*
Before 1960	19	1.00	0.89	0.84
1960–1979	33	1.00	0.67	0.85
1980–1999	106	0.99	0.58	0.70
2000 or later	127	0.96	0.48	0.64

### Effect of Photoperiod Alleles on Heading Date

Heading date had a grand mean of 130.8 days among all 299 entries and nine environments. In the combined analysis *Ppd-A1* had a significant effect (*P* < 0.001) on heading date across environments. Entries with the photoperiod sensitive allele *Ppd-A1b* reached heading an average of 2.0 days later than those with *Ppd-A1a* (S3 Table). The effect of *Ppd-A1* was not significantly different among environments, indicating a lack of significant genotype-by-environment (G×E) interaction at *Ppd-A1*. However, in analyses of individual environments the effect was significant (*P* < 0.05) only at Ar13R, where the effect size was 5.0 days. Only six entries had *Ppd-A1a* and the lack of significant effects in most environments is likely influenced by low diversity at this locus.

The effect of *Ppd-B1* on heading date was significant in the combined analysis (*P* < 0.001). *Ppd-B1b* was associated with 3.0 days later heading across environments, but there was significant G×E interaction. *Ppd-B1* had a significant effect (*P* < 0.01, S3 Table) in all individual environments. The effect of *Ppd-B1b* ranged from a minimum of 0.8 days later heading date in Fo13 (latitude of 40.65°N, Table 1) to a maximum of 5.2 days later in Ar13R (latitude of 34.18°N).

There was a significant effect (*P* < 0.001) of *Ppd-D1* on heading date in the combined analysis. The average effect of *Ppd-D1b* was 3.2 days later heading, which was larger than the effects of *Ppd-B1b* or *Ppd*-*A1b*. The effect of *Ppd-D1* was significant (*P* < 0.05) in six environments, and the effect size ranged from a minimum of 0.9 day in Fo13 to a maximum of 5.2 days in Ar13R and It12R (S3 Table).

**Fig 1:**
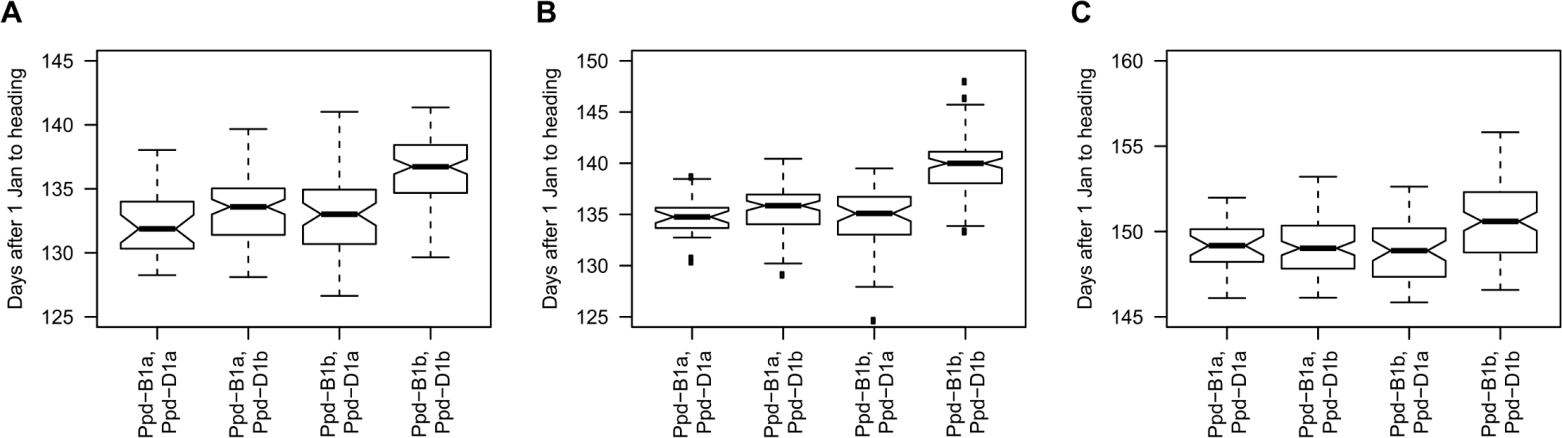
Box plot of number of days from 1 January to heading of 299 hard winter wheat entries varying for photoperiod insensitive (*Ppd-B1a*, *Ppd-D1a*) and sensitive alleles (*Ppd-B1b*, *Ppd-D1b*), evaluated in four Colorado environments. The box describes the minimum, lower quartile (25th percentile), median (50th percentile), upper quartile (75th percentile) values. The notch displays the 95% confidence interval around the median value, and if the notches don’t overlap between two boxes on the same plot, there is strong evidence their median values differ. The interquartile range is described as the upper quartile minus the lower quartile. The whiskers extend to the most extreme data point that is up to 1.5 times the interquartile range from the median value. Outlying points that fall outside of this range are represented as dots. The environments are (A) partial irrigation at Greeley, CO in 2012 (Gr12P), (B) full irrigation at Greeley, CO in 2012 (Gr12F), and (C) Fort Collins, CO in 2013 (Fo13).

Models including *Ppd-D1*, *Ppd-B1*, and the interaction between *Ppd-D1* and *Ppd-B1* explained up to 65% of the variability in heading date (S4 Table). The interaction between *Ppd-B1* and *Ppd-D1* was significant in most environments (excluding Ha13R), but when the interaction was included the main effects of the loci generally became non-significant (S4 Table). The interaction was such that entries with both photoperiod sensitive alleles reached heading much later than those with a single photoperiod sensitive allele or both photoperiod insensitive alleles. The effect of carrying both photoperiod sensitive alleles was greater than 6 days in four environments: Ar13R (8.6 days), It12R (8.0 days), Bu12R (6.7 days), and Ma12 (6.4 days). The effect of the significant interaction between *Ppd-B1* and *Ppd-D1* is illustrated for three Colorado environments (Gr12P, Gr12F, and Fo13) in Fig 1A–1C. The effect of interaction between *Ppd-D1b* and *Ppd-B1b* in the Colorado environments ranged from 1.5 to 4.1 days (S4 Table).

### Effect of Semi-Dwarf Alleles on Heading Date

In the combined analysis, the semi-dwarf allele *Rht-B1b* had a significant (*P* < 0.001) effect on heading date and significant (*P* < 0.001) G×E interaction. *Rht-B1b* was associated with an average effect of 2.4 days earlier heading, but the effect ranged from 1.1 days in Ha13R to 4.2 days in Ar13R (S3 Table). There was a low level of genetic diversity at *Rht-D1* and the locus did not have a significant effect on heading date when it was the only genetic term in the model.

There were 289 entries with homozygous allele calls at *Rht-B1* and *Rht-D1*. When both loci were included in the model, the main effects of both *Rht-B1* and *Rht-D1* were significant (*P* < 0.001, Table 5). There was significant G×E interaction for *Rht-B1* but not *Rht-D1*. Across environments *Rht-D1b* had an effect of 2.9 days earlier heading. The effect of *Rht-B1b* was significant (*P* < 0.05) in Ar13R, Bu12R, Gr12P, and It12R and ranged from a minimum of 2.6 days earlier heading in Gr12P to a maximum of 4.7 days earlier in Ar13R (Table 6). Interaction between *Rht-B1* and *Rht-D1* could not be tested because not all four allelic combinations were present in this germplasm.

**Table 5:** ANOVA table for two-gene model estimating the effects of *Rht-B1* and *Rht-D1* on heading date in a combined analysis across nine environments. A total of 285 entries with homozygous calls at both loci were included in the analysis.

Source of variation	df	Mean Square
Environment	8	55935***
*Rht-B1*	1	3838***
*Rht-D1*	1	741***
Environment*Rht-B1	8	73.8***
Error	2546	13.9

*** indicates significance at the 0.001 probability level.

**Table 6:** Allelic effects (number of days) of *Rht-B1b* in a model that estimated days after 1 January to heading in 285 winter wheat entries grown in nine environments. The environments are described in Table 1. The model terms were fit separately for each environment. The model effects included environment, *Rht-B1*, *Rht-D1*, and *Rht-B1*-by-environment interaction. The intercept (Int) describes the number of days from 1 January to heading in each environment before the allelic effects are applied. The allelic effect (number of days) at each locus is added to the Int value. The allelic effect of *Rht-D1b* was -2.68 days and did not have significant interaction with environment.

Env	Int	*Rht-B1b*
Ar13R	112.52	-4.72**
Bu12R	119.76	-4.18*
Fo13	151.02	-1.70 ns^1^
Gr12F	138.38	-2.05 ns
Gr12P	136.40	-2.62***
Ha13R	142.15	-1.63 ns
Ma12	124.53	-2.83 ns
It12R	125.46	-4.14*
It13R	147.75	-2.68 ns

*, **, *** indicates significance at the 0.05, 0.01 or 0.001 probability level.

^1^ ns indicates non-significance at the 0.05 probability level.

### Effect of Vernalization Alleles on Heading Date

No entry had alleles for spring growth habit detected at *Vrn-A1*, *Vrn-B1*, or *Vrn-D1*. The effect of different *vrn-B1* winter alleles was significant (*P* <0.01) in the combined analysis, with the ‘Neuse’ allele heading 2.2 days later (S3 Table). However, only two entries carried the *vrn-B1 ‘*AGS2000’ allele. The G×E term was not significant for *vrn-B1*.

Variation observed for the winter allele *vrn-A1* had a significant effect (*P* < 0.001) on heading date that varied among environments (S3 Table). The differential effect of three or more *vrn-A1* copies relative to a single copy ranged from a minimum of 0.5 days later heading in Gr12P to a maximum of 4.7 days later heading in Ar13R. The effect of three or more copies of *vrn-A1* relative to two copies also had a significant (*P* < 0.01) effect in the combined analysis, such that entries with two copies reached heading 1.9 days earlier. This effect had significant G×E interaction, and in individual environments the effects of three or more copies relative to two copies were only significant in Ar13R, where the effect size was 5.5 days (S3 Table).

Copy number variation at *Vrn-A1* has been previously shown to have a positive association with vernalization requirements and survival under freezing conditions [46]. The strong effect of *vrn-A1* in Ar13R suggests winter temperatures at this environment may not have fully satisfied the vernalization requirements of all entries. Ar13R experienced a mild winter with daily highs always above 0°C. The effect of allelic variation at *Vrn-A1* did not have a significant effect at It12R, which experienced a cold winter with 11 days that had highs below 0°C and 65 days with lows below 0°C.

### Multivariate Analyses of Alleles on Heading Date

#### Combined analyses across environments

The significance of *Ppd-A1*, *Ppd-B1*, *Ppd-D1*, *Rht-B1*, *Rht-D1*, and their interactions were tested in a multiple-gene model using a subset of 280 entries with complete genotypic data and homozygous calls at all loci (Table 3). For the combined analysis the full model included main effects of the environment, main effects of all five genes, all 10 two-way interactions between genes, and the interaction of each genetic term with the environment. Model selection was used to identify the best-fit model based on lowest AIC value.

The best-fit model to explain heading date across environments included effects of the environment; major effects of all five candidate loci (*Ppd-D1*, *Ppd-B1*, *Ppd*-*A1*, *Rht-B1*, and *Rht-D1*); the interaction between *Ppd-D1* and *Ppd-B1*; the interaction between *Ppd-B1* and *Rht-B1*; two-way interactions between the environment and *Ppd-D1*, *Ppd-B1*, and *Rht-B1*; and a three-way interaction between the environment, *Ppd-D1*, and *Ppd-B1* (Table 7). This model explained 96.2% of the phenotypic variation in heading date, with most of the variation (92.2% of sums of squares) explained by the environment, and a smaller amount (4.2%) explained by genetic terms. Of the variation explained by genetic terms, 30.1% was due to the main effect of *Ppd-B1*, 29.6% was due to *Ppd-D1*, and 13.6% was due to their interaction. Smaller amounts of the genetic variation were explained by the two-way interactions between *Ppd-D1* (9.1%) or *Ppd-B1* (8.7%) and the environment, the three-way interaction between *Ppd-D1*, *Ppd-B1*, and the environment (4.1%), the main effect of *Rht-B1* (2.7%), and the interaction between *Rht-B1* and *Ppd-B1* (1.2%). Less than 1% of the genetic variation was due to *Ppd-A1*, *Rht-D1*, the two-way interaction between *Ppd-B1* and *Ppd-A1*, or the two-way interaction between *Rht-B1* and the environment. The relative importance of each locus in explaining variation in heading date was also evaluated through model averaging using Akaike weights. The Akaike weights were 1.00 for *Ppd-B1*, *Ppd-D1*, and *Rht-B1;* 0.96 for *Ppd-A1*; and 0.71 for *Rht-D1*.

**Table 7:** ANOVA table for the best-fit model, considering effects of environment, *Ppd-A1*, *Ppd-B1*, *Ppd-D1*, *Rht-B1*, *Rht-D1*, and their interactions on winter wheat heading date in a combined analysis across nine environments. The sums of squares were used to estimate the proportion of total variance each term contributed, and the proportion of genetic variance each genetic term contributed. A total of 280 entries with homozygous calls at each allele were included in the model.

Source of variation	df	Mean Squares	Proportion of Total Variance	Proportion of Genetic Variance
Environment	8	54154***	0.922	
*Ppd-D1*	1	5807***	0.012	0.296
*Ppd-B1*	1	5891***	0.013	0.301
*Ppd-A1*	1	6 ns	0.000	0.000
*Rht-B1*	1	523***	0.001	0.027
*Rht-D1*	1	16 ns	0.000	0.001
Ppd-B1*Ppd-A1	1	26 ns	0.000	0.001
Ppd-D1*Ppd-B1	1	2674***	0.006	0.136
Ppd-B1*Rht-B1	1	238***	0.001	0.012
Environment*Ppd-D1	8	223***	0.004	0.091
Environment*Ppd-B1	8	214***	0.004	0.087
Environment*Rht-B1	8	15*	0.000	0.006
Environment*Ppd-D1*Ppd-B1	8	100***	0.002	0.041
Error	2471	7	0.037	

*, *** indicates significance at the 0.05 or 0.001 probability level.

#### Individual analyses for each environment

Multi-gene models were fit to each environment separately using the same model selection approach as in the combined analysis. The full model included the main effects and all two-way interactions between five genes: *Ppd-A1*, *Ppd-B1*, *Ppd-D1*, *Rht-B1*, and *Rht-D1*. The best-fit models varied among environments and are summarized in Table 7, and not all genetic terms are significant. The effects of *Ppd-D1*, *Ppd-B1*, and the interaction between *Ppd-D1* and *Ppd-B1* were included in the best-fit model for each environment. The interaction between *Ppd-D1* and *Ppd-B1* had a maximum effect size of 8.5 days in Ar13R. *Rht-B1* was also included for every environment except Gr12F. None of the best-fit models included the main effect of, or interactions with*Ppd-A1* or *Rht-D1*. Some best-fit models also included interactions between *Ppd-B1* and *Rht-B1* or *Ppd-D1* and *Rht-B1*. The combined allelic effects explained an average of 44% of the variability in heading date, but ranged from a minimum of 14.7% in Fo13 to a maximum of 69.5% in Ma12 (Table 8).

**Table 8:** Allelic effects (number of days) of gene-based terms included in the best-fit model for winter wheat heading date in each of nine environments, and the proportion of variability (*R*^*2*^) in heading date explained by all terms in each model. The environments are described in Table 1. The model terms were fit separately for each environment. The intercept (Int) describes the number of days from 1 January to heading in each environment before the allelic effects are applied. The allelic effect (number of days) at each locus is added to the Int value.

Env	Int	*Ppd-D1b*	*Ppd-B1b*	*Rht-B1b*	Ppd-D1b* *Ppd-B1b*	Ppd-D1b* *Rht-B1b*	Ppd-B1b* *Rht-B1b*	*R*^*2*^
Ar13R	107.04***	2.39 ns	5.73***	-1.53 ns^1^	8.49***	-2.26 ns	2.26 ns	0.57
Bu12R	115.85***	1.26 ns	-2.02 ns	-3.11***	6.71***	—	2.14*	0.59
Fo13	149.82***	-0.14 ns	-0.25 ns	-0.87***	1.34**	—	—	0.15
Gr12F	134.68***	0.80 ns	-0.07 ns	—^2^	4.18***	—	—	0.41
Gr12P	134.11***	0.88 ns	-0.51 ns	-2.02**	2.51***	—	1.17	0.31
Ha13R	140.28***	0.43 ns	0.17 ns	-0.57**	1.57***	—	—	0.33
It12R	121.83***	0.83 ns	-3.10**	-3.57***	8.20***	—	3.34***	0.64
It13R	146.38***	-0.22 ns	-1.30 ns	-2.07***	3.42***	—	1.13 ns	0.31
Ma12	120.89***	0.91 ns	-2.03**	-2.00***	6.80***	—	2.49***	0.69

*, **, *** indicated significance at the 0.05, 0.01, and 0.001 probability levels, respectively.

^1^ ns indicates non-significance at the 0.05 probability level.

^2^—indicates term was not included in best-fit model.

### Geographic Distribution of Alleles

We investigated the geographic distribution of alleles at the vernalization, photoperiod, and reduced-height loci among 263 entries derived from public breeding programs in Colorado, Kansas, Montana, Nebraska, North Dakota, Oklahoma, South Dakota, and Texas. Average heading date of these 263 entries, across all nine environments, was 131.0 ± 13.6 days after 1 January, or about 11 May. The distribution of alleles was expected to vary among breeding programs in different states, because the regions for those programs have variable environmental conditions known to affect heading date. For instance, Texas has much milder winter temperatures than northern locations, and Montana and North Dakota have greater changes in day length throughout the year than the central and southern plains, due to their northern latitudes.

**Fig 2:**
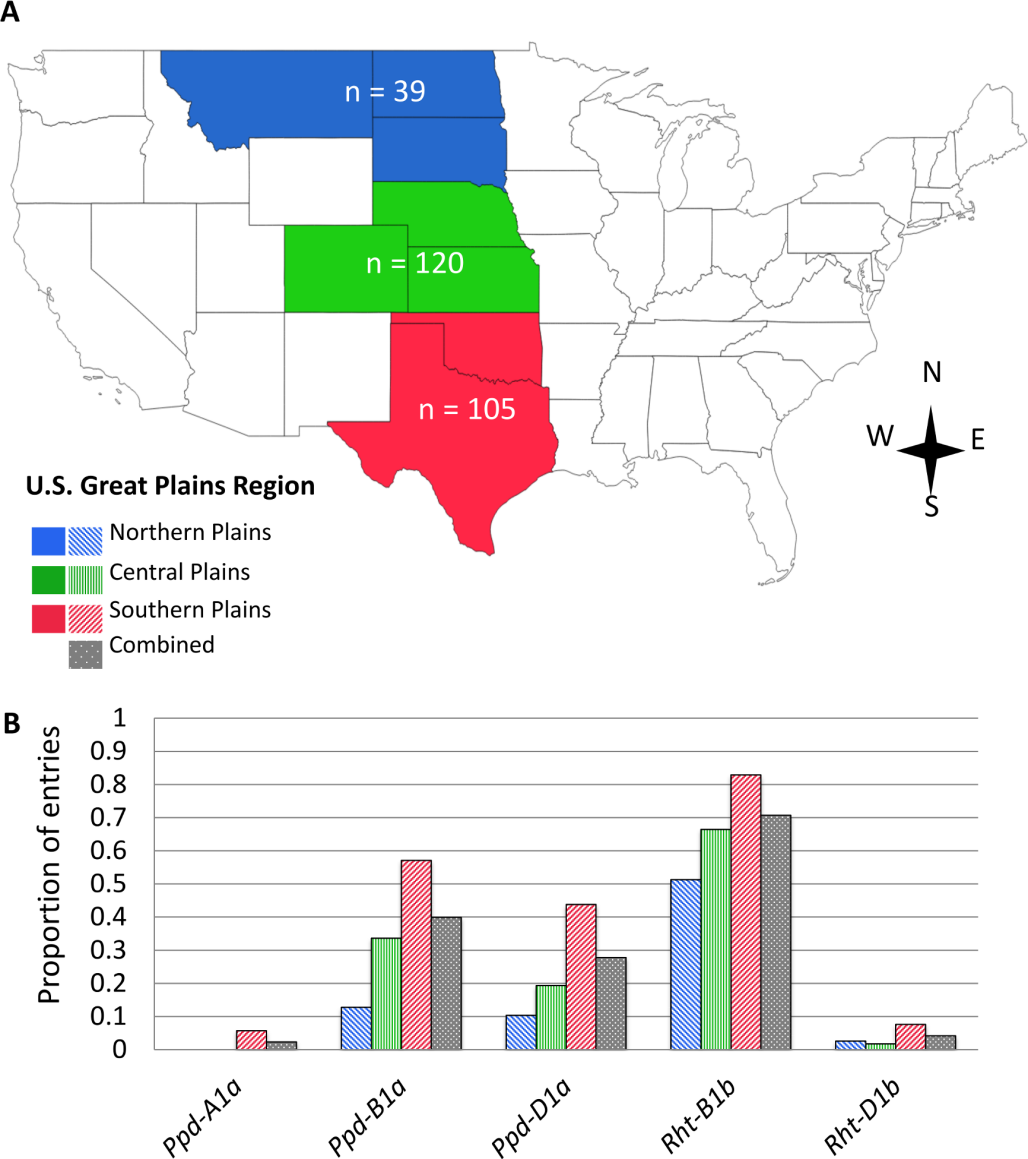
Frequency of photoperiod and reduced-height alleles in wheat entries from three regions of the U.S. Great Plains. Map shapefiles are open source and freely available from Natural Earth < http://www.naturalearthdata.com>. (A) Geographic distribution of 263 winter wheat entries that originated from the northern (Montana, North Dakota, South Dakota, n = 39 entries), central (Nebraska, Colorado, Kansas, n = 119), or southern (Oklahoma, Texas, n = 105) U.S. Great Plains. (B) Proportion of wheat entries from each U.S. Great Plains sub-region, or combined across all three sub-regions, with the photoperiod insensitive alleles *Ppd-A1a*, *Ppd-B1a*, and *Ppd-D1a*, and semi-dwarf alleles *Rht-B1b*, *Rht-D1b*.

In a combined analysis across environments there were significant differences (*P* < 0.001) in heading date among entries from different states of origin. However, not all pairs of states had significantly different heading dates. The entries were divided into three broad regions within the U.S. Great Plains: northern plains (Montana, North Dakota, and South Dakota; 39 entries), southern plains (Texas and Oklahoma; 105 entries), and central plains (Colorado, Kansas, and Nebraska; 119 entries, Fig 2A). There were significant differences (*P* < 0.01) in heading dates among entries originating from each pair of regions. Average heading dates were earliest among entries derived in the southern plains (128.8 ± 14.3 days after 1 January). Heading occurred about 2.8 days later in entries derived in the central plains (131.6 ± 13.1 days) compared to entries from the southern plains. Latest heading dates were observed for germplasm from the northern plains (134.9 ± 12.2 days).

All three photoperiod insensitive alleles (*Ppd-A1a*, *Ppd-B1a*, *Ppd-D1a*) were present at higher levels in germplasm from the southern plains than those from the central or northern plains (Fig 2B). The photoperiod insensitive allele *Ppd-A1a* was uncommon in this germplasm. *Ppd-A1a* was only present in six (2%) of the 263 entries with known geographic origins, all of which originated in the southern plains. *Ppd-B1a* was much more evenly distributed—present in 40% of entries overall—but varied gradually among the three regions. *Ppd-B1a* was found at highest proportions (57%) in germplasm from the southern plains, and at much lower levels in the central (34%) or northern plains (13%). Similar patterns were observed for *Ppd-D1a*, which was found in 44% of entries from the southern plains, 19% of entries from the central plains, and only 10% of entries from the northern plains.

The semi-dwarf alleles at the reduced-height loci were more common in germplasm derived in the southern plains than those originating from the northern or central plains. *Rht-B1b* was found in 71% of entries overall, including 83% of entries from the southern plains, 63% of entries from the central plains, and only 51% of entries from the northern plains (Fig 2B). The semi-dwarf allele *Rht-D1b* was rare in the panel, and present in only 11 (4%) entries. However, the distribution of this allele was much higher in the southern plains (8 entries, 8%) than the northern plains (1 entry, 3%) or central plains (2 entries, 2%).

## Discussion

Our first objective was to characterize allelic variants at loci known to affect developmental timing of wheat through the vernalization, photoperiod sensitivity, and reduced-height pathways. Marker analysis revealed the incidence of semi-dwarf alleles *Rht-B1b* and *Rht-D1b* in this germplasm was comparable to that reported by Guedira et al. [23] in a similar set of germplasm. The photoperiod insensitive alleles *Ppd-B1a* and *Ppd-D1a* were present at similar levels as detected by Kiss et al. [24] in a worldwide collection of winter wheat. We found *Ppd-A1a* to be rare in this germplasm, and present at slightly lower levels than reported among European accessions [26]. Further allelic variation could exist in our panel as additional allelic variants [40, 50, 64] or copy number variants [25, 65] not detected by our assays.

Guo et al. [40] described four modern and two ancient *Ppd-D1* haplotypes present in a worldwide collection of wheat, and detected all four modern haplotypes in accessions from the U.S. and Canada. Our analyses could only differentiate two modern and one ancient haplotype, which suggests additional haplotypes could be present. Seven of our entries had an alternate photoperiod sensitivity allele at *Ppd-D1b*, described by Guo et al. [40] as haplotype VI. The alternate allele did not have a significant effect on heading date across environments, probably due to the small sample size. The entries with haplotype VI are ‘OK Rising’ (PI 656382), ‘Thunder CL’ [66], TAM 303, KS00F5-20-3, ‘Overley’ (PI 634974), ‘Chisholm’ [67], and ‘Custer’ (OK88767-11). Haplotype VI is thought to be an ancient deletion and is associated with *Aegilops tauschii* accessions and synthetic hexaploid wheats [40]. All seven entries we characterized as haplotype VI have pedigree contributions from *Aegilops tauschii* or Eastern European germplasm. The Eastern European parentage might also include *Aegilops tauschii*, as exotic germplasm and interspecific crosses were introduced to this region during the 1970s and 1980s [68].

Additional alleles have also been described at *Ppd-A1* [50]. The photoperiod insensitive allele *Ppd-A1a* was expected to be associated with earlier heading, but did not have a significant effect on heading date on this germplasm. We only characterized six entries as carrying *Ppd-A1a*, all of which originated from the southern plains. This region has less variation in day length than the other U.S. Great Plains regions, so photoperiod sensitivity is expected to have less of an effect, and photoperiod insensitivity to be more common, than at more northern latitudes. Guedira et al. [49] found the photoperiod insensitive allele *Ppd-A1a*.*1*—first identified in Japanese germplasm—to be common among winter wheat varieties originating in the eastern U.S. [50]. It is likely that the effect of *Ppd-A1a* on heading date would be significant among germplasm with greater diversity at this locus, such as a panel that included more entries from southern or eastern regions of the United States.

Our second objective was to estimate the allelic effects of known developmental genes on heading date. We found the timing of heading date in winter wheat from the U.S. Great Plains to be more strongly affected by photoperiod loci than the vernalization or reduced-height loci. Alleles of these five developmental genes (*Ppd-A1*, *Ppd-B1*, *Ppd-D1*, *Rht-B1*, and *Rht-D1*) had the greatest genetic effects on variation in heading date (Table 7). However, terms included in the best-fit model varied slightly among environments (Table 8). Additionally, the effects of *Ppd-A1* and *Rht-D1* were not significant in any individual environment, and have relatively low Akaike weights, which indicates a lower level of likelihood these loci are affecting heading date. Differential effects among environments suggest the model might be improved if the environmental effects were deconstructed into multiple environmental variables (such as effects of temperature, moisture, day length, etc.). Furthermore, additional variation in number of days after 1 January to heading might be improved through genotyping and inclusion of other loci known to affect developmental timing, such as *Vrn-B3* and *Vrn-D3* [11, 19, 45].

In our study, *Rht-B1* was found to have a significant effect on heading date in most environments (Table 8). While it is unclear how reduced sensitivity to GA affects heading date, *Rht-B1* has been previously shown to have a small effect on heading date [20]. Wilhelm et al. [69] suggest one possibility is the role of other genes that are tightly linked with *Rht-B1*, such as *Teosinte branched 1* (*TaTb1*), which is associated with tillering and fertility. Recently, Li. et al. [64] reported numerous allelic variants at *Rht1 (Rht-A1*, *Rht-B1*, and *Rht-D1)*, which were largely unaccounted for in our analyses. Among these variants were novel alleles that increased plant height relative to the tall alleles *Rht-B1a* or *Rht-D1a*.

Most of the genetic effects were due to the main effects and interaction of *Ppd-B1* and *Ppd-D1*, and their three-way interaction with the environment. The interaction between *Ppd-B1* and *Ppd-D1* was such that entries carrying both photoperiod sensitive alleles reached heading much later than those carrying a single photoperiod sensitive allele or both insensitive alleles (Fig 1, S4 Table, Table 8). The presence of both photoperiod sensitive alleles delayed heading date by an average of 4.8 days across all environments, and a maximum of 8.5 days in Ar13R (Table 8). While the genetic effects on heading date are small relative to the environmental effects (Table 7), even minor variation in the timing of heading date—variation of several days—may allow for more precise targeting of wheat varieties to different regions, especially if climatic patterns shift within established growing regions [70], or wheat cultivation expands to new regions [71].

The allelic effects of *Ppd-B1* and *Ppd-D1* varied among environments, suggesting differential expression under varying environmental conditions such as temperature, or moisture. The greatest significant effects—more than five days at each locus—were observed at Ar13R (S3 Table). The effect of the interaction between these loci was 8.6 days at Ar13R (S4 Table). Variation in the effect size and level of significance does not follow a discernable trend among environments; however, the G×E term is likely affected by different sources of environmental variability, such as climatic conditions, management practices including planting and harvest dates, or biotic and abiotic stresses. For instance, heavy rainfall during May and June, and warm maximum daily temperatures throughout the winter, spring, and summer have influenced the large allelic effects at Ar13R. Interaction between *Ppd-D1* and *Ppd-B1* has been previously characterized [72], is known to vary with CNV at *Ppd-B1* [46], and is known to have much larger effects in spring than winter wheat [24].

Allelic variation associated with CNV at *Vrn-A1* had a significant effect on heading date in this germplasm (S3 Table). Low copy number at *Vrn-A1* is associated with earlier flowering following a short vernalization period [16]. Copy number variation at *Vrn-A1* was inferred by SNPs on exons four and seven. The SNP on exon four is associated with having two or fewer copies of the gene, and was used by Eagles et al. [46] to distinguish *Vrn-A1v* associated with earlier heading from *Vrn-A1w* associated with greater freezing tolerance. We found the effect of CNV at *Vrn-A1* to vary among environments with contrasting winter temperatures. The effect of CNV at *vrn-A1* was largest (4.7 days) at Ar13R (S3 Table), and that environment experienced a mild winter that had daily highs above 0°C and only three days with lows below 0°C. The second largest significant effect was seen at Bu12R, which is also located in a mild, southern environment. By contrast, variation at *Vrn-A1* did not have a significant effect at It12R, which experienced a cold winter with 65 days—including 29 continuous days—that had lows below 0°C.

Our third objective was to investigate temporal and geographic patterns of allelic distribution in the U.S. Great Plains. Changes in allele frequencies at the photoperiod genes indicate selection for photoperiod insensitivity during the last century of winter wheat breeding (Table 4). This could be the direct result of selecting for improved adaptability based on flowering time [73]. Most strikingly, the proportion of entries carrying the *Ppd-B1b* sensitive allele has gradually declined over time, from 89% of entries derived before 1960 to only 48% of germplasm derived after 2000 (Table 4). An increase in the number of varieties carrying the photoperiod insensitive allele suggests a contribution to greater adaptation to specific environments.

Selection against photoperiod sensitivity is also apparent in the distribution of alleles from breeding programs in different sub-regions of the U.S. Great Plains. We saw the incidence of photoperiod sensitive alleles decrease from northern to southern latitudes (Table 8). Various environmental variables, such as temperature and day length, vary with latitude. Extreme temperature events such as freezing temperatures and heat stress can have very pronounced and detrimental effects on yield, especially during vulnerable developmental stages. Therefore, targeting heading date could reduce the chance that extreme temperatures or stress damage the developing reproductive structures.

## Conclusions

In this study we used a panel of 299 hard winter wheat entries representative of modern and historic U.S. Great Plains germplasm to evaluate allelic diversity and effects of vernalization, photoperiod, and reduced-height loci on the timing of heading date. We found most of the genetic effects of heading date to be explained by *Ppd-B1*, *Ppd-D1*, and the interaction between these loci. A smaller amount of variation was explained by *Rht-B1*. Across nine environments, the interaction between *Rht-B1* and *Ppd-B1* also had a small but significant effect on heading date. Both photoperiod sensitive and insensitive alleles were common for *Ppd-B1* and *Ppd-D1*, and an alternate photoperiod sensitivity allele associated with ancestral wheats was detected at *Ppd-D1*. There was limited allelic diversity at *Ppd-A1* and *Rht-D1*, and these loci did not have a significant effect on heading date. The presence of photoperiod sensitive alleles *Ppd-A1b*, *Ppd-B1b*, and *Ppd-D1b* has been decreasing over the past century of wheat breeding, and these alleles are less common in the southern sub-region of the U.S. Great Plains than either the central or northern plains. Our analyses enhance the understanding of the effects of developmental genes on winter wheat under varying environmental conditions. This information can potentially be used to improve breeding strategies for current and future climate scenarios.

## Supporting Information

S1 Table Description of U.S. winter wheat entries evaluated, including entry name, year derived, state breeding program and region of the U.S. Great Plains in which derived, and allele calls. The table is sorted first alphabetically by region, then temporally by year, and finally alphabetically by entry name. The genotyped loci include photoperiod (*Ppd*) genes where alleles are ‘*a*’ insensitive and ‘*b*’ sensitive, reduced-height (*Rht*) genes where alleles are ‘*a*’ tall and ‘*b*’ semi-dwarf, and vernalization (*Vrn*) genes. *Vrn-A1*, *Vrn-B1*, and *Vrn-D1* allele calls are ‘*W*’ for winter growth habit. Variation in winter alleles at vernalization loci include copy number variation (CNV) for *vrn-A1*, Wichita-type (‘*w*’) or Veery-type (‘v’) alleles for *vrn-A1*, and Neuse-type (‘*N*’) or AGS2000-type (‘*A*’) alleles for *vrn-B1*. Missing genotypic data are indicated with a dash (-).
(DOCX)

S2 Table Description of KASP markers used to genotype 299 U.S. Great Plains hard winter wheat entries. KASP detected allelic variants at *Vrn-A1*, *Vrn-B1*, *Vrn-D1*, *Ppd-A1*, *Ppd-B1*, *Ppd-D1*, *Rht-B1*, and *Rht-D1*.
(DOCX)

S3 Table Allelic effects (number of days) of photoperiod, reduced-height, and vernalization loci on heading date in each of nine environments. The environments are described in Table 1. The model terms were fit separately for each locus. The intercept (Int) describes the number of days from1 January to heading in each environment before the allelic effect is applied. The allelic effect at each locus is added to the Int value. Allelic effects were fit separately for each environment when there was significant genotype-by-environment (G×E) interaction at that locus in the combined analysis across all environments. Allelic effects from the combined analyses are reported for *Ppd-A1* and *vrn-B1* because significant G×E interaction was not observed at these loci. The grand mean heading date across all germplasm and environments was 131.8 ± 0.3 days.
(DOCX)

S4 Table Allelic effects (number of days) of photoperiod sensitive alleles *Ppd-D1b* and *Ppd-B1b*. Allelic effects and interaction of *Ppd-D1b* and *Ppd-B1b* on winter wheat heading date in each of nine environments, and proportion of variability (*R*^*2*^) in heading date explained by all terms in each model. The environments are described in Table 1. The model terms were fit separately for each environment. The intercept (Int) describes the number of days from 1 January to heading in each environment before the allelic effects are applied. The allelic effect (number of days) at each locus is added to the Int value.
(DOCX)
