# Supplementary material for: Allelic Variation in Developmental Genes and Effects on Winter Wheat Heading Date in the U.S. Great Plains
Source: PLoS One. 2016 Apr 8;11(4):e0152852. doi: 10.1371/journal.pone.0152852 (PMC4825937; doi:10.1371/journal.pone.0152852)
Supplement: S1 Table — The table is sorted first alphabetically by region, then temporally by year, and finally alphabetically by entry name. The genotyped loci include photoperiod (Ppd) genes where alleles are ‘a’ insensitive and ‘b’ sensitive, reduced-height (Rht) genes where alleles are ‘a’ tall and ‘b’ semi-dwarf, and vernalization (Vrn) genes. Vrn-A1, Vrn-B1, and Vrn-D1 allele calls are ‘W’ for winter growth habit. Variation in winter alleles at vernalization loci include copy number variation (CNV) for vrn-A1, Wichita-type (‘w’) or Veery-type (‘v’) alleles for vrn-A1, and Neuse-type (‘N’) or AGS2000-type (‘A’) alleles for vrn-B1. Missing genotypic data are indicated with a dash (-). (DOCX) [file pone.0152852.s001.docx]

**S1 Table.** **Description of U.S. winter wheat entries evaluated,** including entry name, year derived, state breeding program and region of the U.S. Great Plains in which derived, and allele calls. The table is sorted first alphabetically by region, then temporally by year, and finally alphabetically by entry name. The genotyped loci include photoperiod (*Ppd*) genes where alleles are ‘*a*’ insensitive and ‘*b*’ sensitive, reduced-height (*Rht*) genes where alleles are ‘*a*’ tall and ‘*b*’ semi-dwarf, and vernalization (*Vrn*) genes. *Vrn-A1*, *Vrn-B1*, and *Vrn-D1* allele calls are ‘*W*’ for winter growth habit. Variation in winter alleles at vernalization loci include copy number variation (CNV) for *vrn-A1*, Wichita-type (‘*w*’) or Veery-type (‘v’) alleles for *vrn-A1*, and Neuse-type (‘*N*’) or AGS2000-type (‘*A*’) alleles for *vrn-B1*. Missing genotypic data are indicated with a dash (-).

| **Entry Name** | **Year Derived** | **State^†^** | **Region**^‡^ | ***Ppd-A1***^¶^ | ***Ppd-B1*** | ***Ppd-D1*** | ***Rht-B1*** | ***Rht-D1*** | ***Vrn-A1*** | ***Vrn-B1*** | ***Vrn-D1*** | **CNV *vrn-A1*** | ***vrn-A1*** | ***vrn-B1*** |
| --- | --- | --- | --- | --- | --- | --- | --- | --- | --- | --- | --- | --- | --- | --- |
| AGATE | 1969 | NE | Central | b | b | b | a | a | W | W | W | > 2 | w | N |
| AKRON | 1988 | CO | Central | b | b | b | b | a | W | W | W | > 2 | w | N |
| ALLIANCE | 1988 | NE | Central | b | b | b | b | a | W | W | W | > 2 | w | N |
| ANTON | 1998 | NE | Central | b | b | b | b | a | W | W | W | > 2 | w | N |
| ARAPAHOE | 1982 | NE | Central | b | b | b | b | a | W | W | W | > 2 | w | N |
| BENNETT | 1973 | NE | Central | b | b | b | a | a | W | W | W | > 2 | w | N |
| BISON | 1946 | KS | Central | b | b | b | a | a | W | W | W | > 2 | w | N |
| BUCKSKIN | 1968 | NE | Central | b | b | b | a | a | W | W | W | > 2 | w | N |
| CAMELOT | 2001 | NE | Central | b | b | b | b | a | W | W | W | > 2 | w | N |
| CARSON | 1981 | CO | Central | b | b | b | a | a | W | W | W | > 2 | w | N |
| CHENEY | 1973 | KS | Central | b | b | b | a | a | W | W | W | > 2 | w | N |
| CHEYENNE | 1922 | NE | Central | b | b | b | a | a | W | W | W | > 2 | w | N |
| CO03W054 | 2003 | CO | Central | b | b | b | b | a | W | W | W | 1 | v | N |
| CO04025 | 2004 | CO | Central | b | b | b | b | a | W | W | W | > 2 | w | N |
| CO04393 | 2004 | CO | Central | b | b | b | b | a | W | W | W | > 2 | w | N |
| CO04W320 | 2004 | CO | Central | b | b | b | b | a | W | W | W | 1 | v | N |
| CO07W245 | 2007 | CO | Central | b | b | b | a | a | W | W | W | > 2 | w | N |
| CO940610 | 1994 | CO | Central | b | b | b | b | a | W | W | W | > 2 | w | N |
| COLT | 1978 | NE | Central | b | b | b | b | a | W | W | W | > 2 | w | N |
| COMANCHE | 1934 | KS | Central | b | b | b | a | a | W | W | W | > 2 | w | N |
| COUGAR | 1993 | NE | Central | b | b | b | a | a | W | W | W | > 2 | w | N |
| DODGE | 1982 | KS | Central | b | b | b | b | a | W | W | W | 1 | v | N |
| EAGLE | 1967 | KS | Central | b | b | b | a | a | W | W | W | > 2 | w | N |
| GAGE | 1952 | NE | Central | b | b | b | a | a | W | W | W | > 2 | w | N |
| GOODSTREAK | 1997 | NE | Central | b | b | b | a | a | W | W | W | > 2 | w | N |
| HAIL | 1977 | CO | Central | b | b | b | a | b | W | W | W | > 2 | w | N |
| HALLAM | 1998 | NE | Central | b | b | b | b | a | W | W | W | > 2 | w | N |
| HOMESTEAD | 1968 | NE | Central | b | b | b | a | a | W | W | W | > 2 | w | N |
| INFINITY_CL | 2001 | NE | Central | b | b | b | b | a | W | W | W | > 2 | w | N |
| JULES | 1986 | CO | Central | b | b | b | a | b | W | W | W | > 2 | w | N |
| KAW61 | 1947 | KS | Central | b | b | b | a | a | W | W | W | > 2 | w | N |
| KIOWA | 1943 | KS | Central | b | b | b | a | a | W | W | W | > 2 | w | N |
| LANCER | 1957 | NE | Central | b | b | b | a | a | W | W | W | > 2 | w | N |
| LARNED | 1970 | KS | Central | b | b | b | a | a | W | W | W | > 2 | w | N |
| MCGILL | 2001 | NE | Central | b | b | b | b | a | W | W | W | > 2 | w | N |
| MILLENNIUM | 1994 | NE | Central | b | b | b | a | a | W | W | W | 1 | v | N |
| NE02558 | 2002 | NE | Central | b | b | b | b | a | W | W | W | 1 | v | N |
| NE04490 | 2004 | NE | Central | b | b | b | a | a | W | W | W | > 2 | w | N |
| NE05496 | 2005 | NE | Central | b | b | b | b | a | W | W | W | > 2 | w | N |
| NE05548 | 2005 | NE | Central | b | b | b | a | a | W | W | W | > 2 | w | N |
| NE06545 | 2006 | NE | Central | b | b | b | b | a | W | W | W | > 2 | w | N |
| NE06607 | 2006 | NE | Central | b | b | b | b | a | W | W | W | > 2 | w | N |
| NE99495 | 1999 | NE | Central | b | b | b | b | a | W | W | W | > 2 | w | N |
| NEKOTA | 1988 | NE | Central | b | b | b | b | a | W | W | W | > 2 | w | N |
| NEWTON | 1973 | KS | Central | b | b | b | b | a | W | W | W | 1 | v | N |
| NIOBRARA | 1989 | NE | Central | b | b | b | a | a | W | W | W | > 2 | w | N |
| NUPLAINS | 1994 | NE | Central | b | b | b | b | a | W | W | W | > 2 | w | N |
| NW03666 | 2003 | NE | Central | b | b | b | b | a | W | W | W | > 2 | w | N |
| OVERLAND | 2001 | NE | Central | b | b | b | b | a | W | W | W | > 2 | w | N |
| REDLAND | 1985 | NE | Central | b | b | b | b | a | W | W | W | > 2 | w | N |
| RIPPER | 2000 | CO | Central | b | b | b | b | a | W | W | W | > 2 | w | N |
| SAGE | 1970 | KS | Central | b | b | b | a | a | W | W | W | > 2 | w | N |
| SCOUT66 | 1956 | NE | Central | b | b | b | a | a | W | W | W | > 2 | w | N |
| VISTA | 1987 | NE | Central | b | b | b | b | a | W | W | W | > 2 | w | N |
| WAHOO | 1994 | NE | Central | b | b | b | a | a | W | W | W | > 2 | w | N |
| WARRIOR | 1948 | NE | Central | b | b | b | a | a | W | W | W | > 2 | w | N |
| WESLEY | 1995 | NE | Central | b | b | b | b | a | W | W | W | > 2 | w | N |
| WICHITA | 1927 | KS | Central | b | b | b | a | a | W | W | W | > 2 | w | N |
| 2145 | 1997 | KS | Central | b | b | a | b | a | W | W | W | > 2 | w | N |
| ABOVE | 1998 | CO | Central | b | b | a | b | a | W | W | W | 2 | v | N |
| ARLIN | 1992 | KS | Central | b | b | a | b | a | W | W | W | > 2 | w | N |
| CULVER | 1993 | NE | Central | b | b | a | a | a | W | W | W | > 2 | w | N |
| DENALI | 2005 | CO | Central | b | b | a | a | b | W | W | W | > 2 | w | N |
| KARL_92 | 1983 | KS | Central | b | b | a | b | a | W | W | W | > 2 | w | N |
| LAKIN | 1996 | KS | Central | b | b | a | b | a | W | W | W | > 2 | w | N |
| NI06736 | 2006 | NE | Central | b | b | a | b | a | W | W | W | > 2 | w | N |
| NI06737 | 2006 | NE | Central | b | b | a | b | a | W | W | W | > 2 | w | N |
| ROBIDOUX | 2004 | NE | Central | b | b | a | b | a | W | W | W | > 2 | w | N |
| SIOUXLAND | 1986 | NE | Central | b | b | a | a | a | W | W | W | > 2 | w | N |
| YUMAR | 1994 | CO | Central | b | b | a | b | a | W | W | W | 2 | v | N |
| BOND_CL | 2000 | CO | Central | b | a | b | b | a | W | W | W | > 2 | w | N |
| BYRD | 2006 | CO | Central | b | a | b | a | a | W | W | W | > 2 | w | N |
| CENTURA | 1977 | NE | Central | b | a | b | a | a | W | W | W | > 2 | w | N |
| CENTURK78 | 1969 | NE | Central | b | a | b | a | a | W | W | W | > 2 | w | N |
| CO03064 | 2003 | CO | Central | b | a | b | b | a | W | W | W | > 2 | w | N |
| CO050337-2 | 2005 | CO | Central | b | a | b | b | a | W | W | W | > 2 | w | N |
| DANBY | 2002 | KS | Central | b | a | b | b | a | W | W | W | > 2 | w | N |
| DUKE | 1974 | CO | Central | b | a | b | a | a | W | W | W | > 2 | w | N |
| FULLER | 2000 | KS | Central | b | a | b | b | a | W | W | W | > 2 | w | N |
| HEYNE | 1985 | KS | Central | b | a | b | b | a | W | W | W | 1 | v | N |
| JAGGER | 1984 | KS | Central | b | a | b | b | a | W | W | W | 1 | v | N |
| KIRWIN | 1966 | KS | Central | b | a | b | a | a | W | W | W | > 2 | w | N |
| KS00F5-20-3 | 2000 | KS | Central | b | a | b | b | a | W | W | W | 1 | v | N |
| LAMAR | 1982 | CO | Central | b | a | b | a | a | W | W | W | > 2 | w | N |
| MACE | 2002 | NE | Central | b | a | b | b | a | W | W | W | > 2 | w | N |
| NI07703 | 2007 | NE | Central | b | a | b | b | a | W | W | W | > 2 | w | N |
| OVERLEY | 1994 | KS | Central | b | a | b | b | a | W | W | W | 1 | v | N |
| PARKER | 1953 | KS | Central | b | a | b | b | a | W | W | W | 1 | v | N |
| PARKER76 | 1974 | KS | Central | b | a | b | a | a | W | W | W | > 2 | w | N |
| PLATTE | 1989 | CO | Central | b | a | b | b | a | W | W | W | > 2 | w | N |
| PRAIRIE_RED | 1994 | CO | Central | b | a | b | b | a | W | W | W | > 2 | w | N |
| PRONGHORN | 1988 | NE | Central | b | a | b | a | a | W | W | W | > 2 | w | N |
| PROWERS | 1994 | CO | Central | b | a | b | a | a | W | W | W | > 2 | w | N |
| RONL | 2003 | KS | Central | b | a | b | b | a | W | W | W | > 2 | w | N |
| SETTLER_CL | 2003 | NE | Central | b | a | b | b | a | W | W | W | > 2 | w | N |
| SHAWNEE | 1960 | KS | Central | b | a | b | a | a | W | W | W | > 2 | w | N |
| STANTON | 1995 | KS | Central | b | a | b | b | a | W | W | W | > 2 | w | N |
| TAM107-R7 | 1994 | NE | Central | b | a | b | b | a | W | W | W | > 2 | w | N |
| TRISON | 1965 | KS | Central | b | a | b | a | a | W | W | W | > 2 | w | N |
| WINDSTAR | 1990 | NE | Central | b | a | b | b | a | W | W | W | 1 | v | N |
| BILL_BROWN | 2001 | CO | Central | b | a | a | b | a | W | W | W | 2 | v | N |
| HALT | 1991 | CO | Central | b | a | a | b | a | W | W | W | > 2 | w | N |
| LINDON | 1972 | CO | Central | b | a | a | b | a | W | W | W | > 2 | w | N |
| NE05430 | 2005 | NE | Central | b | a | a | b | a | W | W | W | > 2 | w | N |
| NI08707 | 2008 | NE | Central | b | a | a | b | a | W | W | W | > 2 | w | N |
| NI08708 | 2008 | NE | Central | b | a | a | b | a | W | W | W | > 2 | w | N |
| RAWHIDE | 1983 | NE | Central | b | a | a | b | a | W | W | W | > 2 | w | N |
| TREGO | 1995 | KS | Central | b | a | a | b | a | W | W | W | > 2 | w | N |
| VONA | 1972 | CO | Central | b | a | a | b | a | W | W | W | > 2 | w | N |
| YUMA | 1985 | CO | Central | b | a | a | b | a | W | W | W | 2 | v | N |
| ANTELOPE | 1997 | NE | Central | b | - | b | b | a | W | W | W | > 2 | w | N |
| SANDY | 1961 | CO | Central | b | - | b | - | a | W | W | W | > 2 | w | N |
| THUNDER_CL | 2003 | CO | Central | b | - | b | b | a | W | W | W | > 2 | w | N |
| HARRY | 1997 | NE | Central | - | - | b | - | a | W | W | W | > 2 | w | N |
| AVALANCHE | 1994 | CO | Central | - | - | - | - | - | W | W | W | > 2 | w | - |
| CO03W043 | 2003 | CO | Central | - | - | - | - | - | W | W | W | - | - | - |
| CO04499 | 2004 | CO | Central | - | - | - | - | - | W | W | W | > 2 | w | - |
| HATCHER | 1998 | CO | Central | - | - | - | - | - | W | W | W | - | - | - |
| NORKAN | 1982 | KS | Central | - | - | - | - | - | W | W | W | > 2 | w | - |
| BIG_SKY | 1994 | MT | North | b | b | b | b | a | W | W | W | > 2 | w | N |
| BRONZE | 1967 | SD | North | b | b | b | a | a | W | W | W | > 2 | w | N |
| CREST | 1966 | MT | North | b | b | b | a | a | W | W | W | > 2 | w | N |
| DARRELL | 1998 | SD | North | b | b | b | b | a | W | W | W | > 2 | w | N |
| DECADE | 2005 | MT | North | b | b | b | b | a | W | W | W | > 2 | w | N |
| GENOU | 2000 | MT | North | b | b | b | a | a | W | W | W | > 2 | w | N |
| GENT | 1971 | SD | North | b | b | b | a | a | W | W | W | > 2 | w | N |
| HARDING | 1992 | SD | North | b | b | b | a | a | W | W | W | > 2 | w | N |
| HUME | 1956 | SD | North | b | b | b | a | a | W | W | W | > 2 | w | N |
| JERRY | 1992 | ND | North | b | b | b | b | a | W | W | W | > 2 | w | N |
| JUDEE | 2007 | MT | North | b | b | b | b | a | W | W | W | > 2 | w | N |
| JUDITH | 1980 | MT | North | b | b | b | b | a | W | W | W | > 2 | w | N |
| MT0495 | 2004 | MT | North | b | b | b | b | a | W | W | W | > 2 | w | N |
| MT06103 | 2006 | MT | North | b | b | b | b | a | W | W | W | > 2 | w | N |
| MT85200 | 1985 | MT | North | b | b | b | a | a | W | W | W | > 2 | w | N |
| MT9513 | 1995 | MT | North | b | b | b | a | a | W | W | W | > 2 | w | N |
| MT9904 | 1999 | MT | North | b | b | b | a | a | W | W | W | > 2 | w | N |
| MT9982 | 1999 | MT | North | b | b | b | b | a | W | W | W | > 2 | w | N |
| MTS0531 | 2005 | MT | North | b | b | b | a | a | W | W | W | > 2 | w | N |
| NELL | 1973 | SD | North | b | b | b | a | a | W | W | W | > 2 | w | N |
| NUSKY | 1994 | MT | North | b | b | b | b | a | W | W | W | > 2 | w | N |
| RITA | 1973 | SD | North | b | b | b | b | a | W | W | W | > 2 | w | N |
| ROSE | 1972 | SD | North | b | b | b | a | a | W | W | W | > 2 | w | N |
| ROSEBUD | 1974 | MT | North | b | b | b | a | a | W | W | W | > 2 | w | N |
| SD00111-9 | 2000 | SD | North | b | b | b | b | a | W | W | W | > 2 | w | N |
| SD05118 | 2005 | SD | North | b | b | b | b | a | W | W | W | > 2 | w | N |
| SD05210 | 2005 | SD | North | b | b | b | b | a | W | W | W | > 2 | w | N |
| TANDEM | 1989 | SD | North | b | b | b | a | a | W | W | W | > 2 | w | N |
| YELLOWSTONE | 2000 | MT | North | b | b | b | b | a | W | W | W | > 2 | w | N |
| ALICE | 1997 | SD | North | b | b | a | b | a | W | W | W | > 2 | w | N |
| SD01058 | 2001 | SD | North | b | b | a | b | a | W | W | W | > 2 | w | N |
| WENDY | 1997 | SD | North | b | b | a | b | a | W | W | W | > 2 | w | N |
| EXPEDITION | 1997 | SD | North | b | a | b | b | a | W | W | W | > 2 | w | N |
| NORRIS | 2003 | MT | North | b | a | b | a | a | W | W | W | > 2 | w | N |
| SD01237 | 2001 | SD | North | b | a | b | b | a | W | W | W | > 2 | w | N |
| WINOKA | 1966 | SD | North | b | a | b | a | b | W | W | W | > 2 | w | N |
| DAWN | 1970 | SD | North | b | a | a | a | a | W | W | W | > 2 | w | N |
| CRIMSON | 1989 | SD | North | b | - | b | a | a | W | W | W | > 2 | w | N |
| SD05W018 | 2005 | SD | North | - | - | b | - | a | W | W | W | > 2 | w | N |
| BAKERS_WHITE | 1998 | - | Other | b | b | b | b | a | W | W | W | 1 | v | N |
| HONDO | 1995 | - | Other | b | b | b | b | a | W | W | W | > 2 | w | N |
| KHARKOF | 1900 | - | Other | b | b | b | a | a | W | W | W | > 2 | w | N |
| NUHORIZON | 1995 | - | Other | b | b | b | b | a | W | W | W | > 2 | w | N |
| SMOKYHILL | 1999 | - | Other | b | b | b | b | a | W | W | W | > 2 | w | N |
| TURKEY_NEBSEL | 1874 | - | Other | b | b | b | a | a | W | W | W | > 2 | w | N |
| BURCHETT | 1996 | - | Other | b | b | a | b | a | W | W | W | > 2 | w | N |
| COSSACK | 1987 | - | Other | b | b | a | a | a | W | W | W | > 2 | w | N |
| E2041 | 2000 | - | Other | b | b | a | a | b | W | W | W | > 2 | w | N |
| ENHANCER | 1998 | - | Other | b | b | a | b | a | W | W | W | > 2 | w | N |
| HV9W03-1551WP | 2003 | - | Other | b | b | a | b | a | W | W | W | > 2 | w | N |
| HV9W03-1596R | 2003 | - | Other | b | b | a | b | a | W | W | W | > 2 | w | N |
| HV9W05-1280R | 2005 | - | Other | b | b | a | a | a | W | W | W | > 2 | w | N |
| KEOTA | 1998 | - | Other | b | b | a | b | a | W | W | W | > 2 | w | N |
| ONAGA | 1991 | - | Other | b | b | a | b | a | W | W | W | > 2 | w | N |
| W04-417 | 2004 | - | Other | b | b | a | a | b | W | W | W | > 2 | w | N |
| WB411W | 1998 | - | Other | b | b | a | b | a | W | W | W | 1 | v | N |
| CUTTER | 1997 | - | Other | b | a | b | b | a | W | W | W | > 2 | w | N |
| G1878 | 1996 | - | Other | b | a | b | b | a | W | W | W | > 2 | w | N |
| HV9W06-504 | 2006 | - | Other | b | a | b | b | a | W | W | W | 1 | v | N |
| JAGALENE | 1998 | - | Other | b | a | b | b | a | W | W | W | > 2 | w | N |
| LONGHORN | 1988 | - | Other | b | a | b | a | a | W | W | W | > 2 | w | N |
| NEOSHO | 1996 | - | Other | b | a | b | a | a | W | W | W | > 2 | w | N |
| NUFRONTIER | 1994 | - | Other | b | a | b | a | a | W | W | W | > 2 | w | N |
| OGALLALA | 1989 | - | Other | b | a | b | b | a | W | W | W | > 2 | w | N |
| POSTROCK | 1995 | - | Other | b | a | b | b | a | W | W | W | > 2 | w | N |
| SANTA_FE | 2003 | - | Other | b | a | b | b | a | W | W | W | 1 | v | N |
| SHOCKER | 1999 | - | Other | b | a | b | b | a | W | W | W | 1 | v | N |
| SPARTAN | 1994 | - | Other | b | a | b | b | a | W | W | W | > 2 | w | N |
| TARKIO | 1999 | - | Other | b | a | b | b | a | W | W | W | > 2 | w | N |
| THUNDERBOLT | 1995 | - | Other | b | a | b | b | a | W | W | W | > 2 | w | N |
| VENANGO | 2000 | - | Other | b | a | b | b | a | W | W | W | > 2 | w | N |
| DUMAS | 1995 | - | Other | b | a | a | a | b | W | W | W | > 2 | w | N |
| HV906-865 | 2006 | - | Other | b | a | a | b | a | W | W | W | > 2 | w | N |
| HV9W03-1379R | 2003 | - | Other | b | a | a | b | a | W | W | W | 1 | v | N |
| HG-9 | <2000 | - | Other | - | - | - | - | - | W | W | W | > 2 | w | - |
| LOCKETT | 1991 | TX | South | b | b | b | b | a | W | W | W | > 2 | w | N |
| OK07S117 | 2007 | OK | South | b | b | b | b | a | W | W | W | > 2 | w | N |
| OK10119 | 2010 | OK | South | b | b | b | b | a | W | W | W | > 2 | w | N |
| OK1067071 | 2010 | OK | South | b | b | b | het | a | W | W | W | > 2 | w | N |
| TAM105 | 1969 | TX | South | b | b | b | b | a | W | W | W | > 2 | w | N |
| TAM109 | 1987 | TX | South | b | b | b | a | b | W | W | W | > 2 | w | N |
| TAMW-101 | 1965 | TX | South | b | b | b | a | b | W | W | W | > 2 | w | N |
| TASCOSA | 1951 | TX | South | b | b | b | a | a | W | W | W | > 2 | w | N |
| TX04V075080 | 2004 | TX | South | b | b | b | b | a | W | W | W | > 2 | w | N |
| TX06V7266 | 2006 | TX | South | b | b | b | b | a | W | W | W | > 2 | w | N |
| TAM302 | 1991 | TX | South | a | b | b | a | het | W | W | W | > 2 | w | N |
| 2180 | 1987 | TX | South | b | b | a | a | b | W | W | W | > 2 | w | N |
| BILLINGS | 2003 | OK | South | b | b | a | b | a | W | W | W | > 2 | w | N |
| CAPROCK | 1956 | TX | South | b | b | a | b | a | W | W | W | > 2 | w | N |
| CHISHOLM | 1975 | OK | South | b | b | a | b | a | W | W | W | > 2 | w | N |
| CUSTER | 1988 | OK | South | b | b | a | b | a | W | W | W | > 2 | w | N |
| DELIVER | 1998 | OK | South | b | b | a | b | a | W | W | W | > 2 | w | N |
| DUSTER | 1993 | OK | South | b | b | a | b | a | W | W | W | > 2 | w | N |
| ENDURANCE | 1994 | OK | South | b | b | a | a | b | W | W | W | > 2 | w | N |
| GALLAGHER | 2007 | OK | South | b | b | a | b | a | W | W | W | > 2 | w | N |
| INTRADA | 1998 | OK | South | b | b | a | het | a | W | W | W | > 2 | w | N |
| MIT | 1971 | TX | South | b | b | a | b | a | W | W | W | 1 | v | N |
| OK02405 | 2002 | OK | South | b | b | a | b | a | W | W | W | > 2 | w | N |
| OK05312 | 2005 | OK | South | b | b | a | b | a | W | W | W | > 2 | w | N |
| RUBY LEE | 2005 | OK | South | b | b | a | het | het | W | W | W | > 2 | w | N |
| OK06114 | 2006 | OK | South | b | b | a | b | a | W | W | W | > 2 | w | N |
| OK06336 | 2006 | OK | South | b | b | a | b | a | W | W | W | > 2 | w | N |
| OK07231 | 2007 | OK | South | b | b | a | b | a | W | W | W | > 2 | w | N |
| OK08328 | 2008 | OK | South | b | b | a | b | a | W | W | W | > 2 | w | N |
| OK101 | 1995 | OK | South | b | b | a | a | b | W | W | W | > 2 | w | N |
| OK1067274 | 2010 | OK | South | b | b | a | a | a | W | W | W | > 2 | w | N |
| OK1068026 | 2010 | OK | South | b | b | a | b | a | W | W | W | > 2 | w | N |
| OK1070267 | 2010 | OK | South | b | b | a | a | a | W | W | W | 1 | v | N |
| STURDY | 1956 | TX | South | b | b | a | b | a | W | W | W | > 2 | w | N |
| STURDY_2K | 1956 | TX | South | b | b | a | b | a | W | W | W | 1 | v | N |
| TAM304 | 2001 | TX | South | b | b | a | b | a | W | W | W | > 2 | w | N |
| TX00V1131 | 2000 | TX | South | b | b | a | b | a | W | W | W | > 2 | w | N |
| TX03A0148 | 2003 | TX | South | b | b | a | b | a | W | W | W | > 2 | w | N |
| TX04A001246 | 2004 | TX | South | b | b | a | b | a | W | W | W | > 2 | w | N |
| TX06A001281 | 2006 | TX | South | b | b | a | b | a | W | W | W | > 2 | w | N |
| TX06A001386 | 2006 | TX | South | b | b | a | b | a | W | W | W | > 2 | w | N |
| TX96D1073 | 1996 | TX | South | b | b | a | b | a | W | W | W | > 2 | w | N |
| OK05303 | 2005 | OK | South | a | b | a | a | b | W | W | W | > 2 | w | N |
| CENTURY | 1981 | OK | South | b | a | b | b | a | W | W | W | > 2 | w | N |
| GUYMON | 2000 | OK | South | b | a | b | b | a | W | W | W | > 2 | w | N |
| OK_BULLET | 2000 | OK | South | b | a | b | b | a | W | W | W | > 2 | w | N |
| OK_RISING | 2006 | OK | South | b | a | b | b | a | W | W | W | > 2 | w | N |
| OK04505 | 2004 | OK | South | b | a | b | b | a | W | W | W | 1 | v | N |
| OK04507 | 2004 | OK | South | b | a | b | b | a | W | W | W | > 2 | w | N |
| OK05108 | 2005 | OK | South | b | a | b | b | a | W | W | W | > 2 | w | N |
| OK05122 | 2005 | OK | South | b | a | b | b | a | W | W | W | > 2 | w | N |
| OK05204 | 2005 | OK | South | b | a | b | b | a | W | W | W | > 2 | w | N |
| OK05711W | 2005 | OK | South | b | a | b | b | a | W | W | W | > 2 | w | N |
| OK05723W | 2005 | OK | South | b | a | b | b | a | W | W | W | > 2 | w | N |
| OK05830 | 2005 | OK | South | b | a | b | b | a | W | W | W | > 2 | w | N |
| OK06210 | 2006 | OK | South | b | a | b | b | a | W | W | W | 1 | v | N |
| OK09634 | 2009 | OK | South | b | a | b | b | a | W | W | W | 1 | v | N |
| OK1068002 | 2010 | OK | South | b | a | b | b | a | W | W | W | > 2 | w | N |
| OK1070275 | 2010 | OK | South | b | a | b | b | a | W | W | W | > 2 | w | N |
| TAM110 | 1988 | TX | South | b | a | b | b | a | W | W | W | > 2 | w | N |
| TAM111 | 1995 | TX | South | b | a | b | b | a | W | W | W | > 2 | w | N |
| TAM112 | 1998 | TX | South | b | a | b | b | a | W | W | W | > 2 | w | N |
| TAM200 | 1981 | TX | South | b | a | b | b | a | W | W | W | > 2 | w | N |
| TAM202 | 1986 | TX | South | b | a | b | b | a | W | W | W | > 2 | w | N |
| TAM203 | 2001 | TX | South | b | a | b | b | a | W | W | W | 1 | v | N |
| TAM303 | 1998 | TX | South | b | a | b | b | a | W | W | W | > 2 | w | N |
| TAM400 | 1993 | TX | South | b | a | b | b | a | W | W | W | > 2 | w | N |
| TAM401 | 2003 | TX | South | b | a | b | b | a | W | W | W | > 2 | w | A |
| TRIUMPH64 | 1938 | OK | South | b | a | b | a | a | W | W | W | > 2 | w | N |
| TX01A5936 | 2001 | TX | South | b | a | b | b | a | W | W | W | > 2 | w | N |
| TX01V5134RC-3 | 2001 | TX | South | b | a | b | b | a | W | W | W | > 2 | w | N |
| TX02A0252 | 2002 | TX | South | b | a | b | b | a | W | W | W | > 2 | w | N |
| TX03A0563 | 2003 | TX | South | b | a | b | b | a | W | W | W | > 2 | w | N |
| TX04M410164 | 2004 | TX | South | b | a | b | b | a | W | W | W | > 2 | w | N |
| TX05A001188 | 2005 | TX | South | b | a | b | b | a | W | W | W | > 2 | w | N |
| TX05A001822 | 2005 | TX | South | b | a | b | b | a | W | W | W | > 2 | v | A |
| TX05V7259 | 2005 | TX | South | b | a | b | b | a | W | W | W | > 2 | w | N |
| TX06A001263 | 2006 | TX | South | b | a | b | b | a | W | W | W | > 2 | w | N |
| TX07A001279 | 2007 | TX | South | b | a | b | b | a | W | W | W | > 2 | w | N |
| TX07A001318 | 2007 | TX | South | b | a | b | b | a | W | W | W | > 2 | w | N |
| TX07A001420 | 2007 | TX | South | b | a | b | b | a | W | W | W | > 2 | w | N |
| TX86A5606 | 1986 | TX | South | b | a | b | b | a | W | W | W | > 2 | w | N |
| TX86A6880 | 1986 | TX | South | b | a | b | b | a | W | W | W | > 2 | w | N |
| TX86A8072 | 1986 | TX | South | b | a | b | b | a | W | W | W | > 2 | w | N |
| TX99A0153-1 | 1999 | TX | South | b | a | b | b | a | W | W | W | > 2 | w | N |
| OK05134 | 2005 | OK | South | a | a | b | het | a | W | W | W | > 2 | w | N |
| TX04M410211 | 2004 | TX | South | a | a | b | b | a | W | W | W | 1 | v | N |
| TX05V7269 | 2005 | TX | South | a | a | b | b | a | W | W | W | > 2 | w | N |
| TX06A001132 | 2006 | TX | South | a | a | b | b | a | W | W | W | > 2 | w | N |
| 2174-05 | 1997 | OK | South | b | a | a | b | a | W | W | W | > 2 | w | N |
| CENTERFIELD | 2003 | OK | South | b | a | a | b | a | W | W | W | > 2 | w | N |
| GARRISON | 2005 | OK | South | b | a | a | b | a | W | W | W | > 2 | w | N |
| OK04111 | 2004 | OK | South | b | a | a | b | a | W | W | W | > 2 | w | N |
| OK04415 | 2004 | OK | South | b | a | a | b | a | W | W | W | > 2 | w | N |
| OK04525 | 2004 | OK | South | b | a | a | a | b | W | W | W | > 2 | w | N |
| OK05511 | 2005 | OK | South | b | a | a | b | a | W | W | W | > 2 | w | N |
| OK06318 | 2006 | OK | South | b | a | a | b | a | W | W | W | > 2 | w | N |
| OK06319 | 2006 | OK | South | b | a | a | b | a | W | W | W | > 2 | w | N |
| OK102 | 1997 | OK | South | b | a | a | b | a | W | W | W | > 2 | w | N |
| OK1068009 | 2010 | OK | South | b | a | a | a | a | W | W | W | > 2 | w | N |
| PETE | 2003 | OK | South | b | a | a | b | a | W | W | W | > 2 | w | N |
| TX01M5009-28 | 2001 | TX | South | b | a | a | b | a | W | W | W | 1 | v | N |
| TX99U8618 | 1999 | TX | South | b | a | a | b | a | W | W | W | 1 | v | N |
| OK1068112 | 2010 | OK | South | b | - | b | b | a | W | W | W | 1 | v | N |
| TAM107 | 1980 | TX | South | b | - | b | b | a | W | W | W | > 2 | w | N |

^†^ Entries contributed by private industry not specific to a state are marked with a dash (-).
^‡^ Regions of the U.S. Great Plains are defined as northern plains (“North”: Montana, North Dakota, and South Dakota), central plains (“Central”: Colorado, Kansas, Nebraska), southern plains (“South”: Texas, and Oklahoma), and all other contributing states (“Other”).
^¶^ Missing genotypic data are indicated with a dash (-).
