# Supplementary material for: Allelic Variation in Developmental Genes and Effects on Winter Wheat Heading Date in the U.S. Great Plains
Source: PLoS One. 2016 Apr 8;11(4):e0152852. doi: 10.1371/journal.pone.0152852 (PMC4825937; doi:10.1371/journal.pone.0152852)
Supplement: S2 Table — KASP detected allelic variants at Vrn-A1, Vrn-B1, Vrn-D1, Ppd-A1, Ppd-B1, Ppd-D1, Rht-B1, and Rht-D1. (DOCX) [file pone.0152852.s002.docx]

**S2 Table. Description of KASP markers used to genotype 299 U.S. Great Plains hard winter wheat entries**. KASP detected allelic variants at Vrn-A1, Vrn-B1, Vrn-D1, Ppd-A1, Ppd-B1, Ppd-D1, Rht-B1, and Rht-D1. KASP assays do not include tail sequences.

| **Locus** | **Allele(s) assayed** | **Marker ID** | **Primer name** | **Primer Sequence** |
| --- | --- | --- | --- | --- |
| Vrn-A1 | Vrn-A1a | wMAS000033 | Vrn-A1_9K0001_AL2 | GAGTTTTCCAAAAAGATAGATCAATGTAAAC |
|  |  |  | Vrn-A1_9K0001_AL1 | AGAGTTTTCCAAAAAGATAGATCAATGTAAAT |
|  |  |  | Vrn-A1_9K0001_C1 | GTTAGTAGTGATGGTCCAATAATGCCAAA |
|  | Vrn-A1b | wMAS000035 | Vrn-A1b-Marq_AL2 | GTTTTGGCCTGGCCATCCTCA |
|  |  |  | Vrn-A1b-Marq_AL1 | GTTTTGGCCTGGCCATCCTCC |
|  |  |  | Vrn-A1b-Marq_C1 | TATCAGGTGGTTGGGTGAGGACGT |
|  | vrn-A1 exon 4_C/T | vrn-A1exon4 | Vrn-A1_Exon4_F1 | AGGCATCTCATGGGAGAGGATC |
|  |  |  | Vrn-A1_Exon4_F2 | CAGGCATCTCATGGGAGAGGATT |
|  |  |  | Vrn-A1_Exon4_R | CCAGTTGCTGCAACTCCTTGAGATT |
|  | vrn-A1 exon 7_G/A | vrn-A1exon7 | Vrn-A1_Exon7_F1 | TGAGTTTGATCTTGCTGCGCCG |
|  |  |  | Vrn-A1_Exon7_F2 | CTGAGTTTGATCTTGCTGCGCCA |
|  |  |  | Vrn-A1_Exon4_R | CTTCCCCACAGCTCGTGGAGAA |
| Vrn-B1 | Vrn-B1a | Vrn-B1_I_D | Vrn-B1_D_A2 | GGCAGCTAATGTGGGGTAGTCT |
|  |  |  | Vrn-B1_D_C1s | ATTCGTATTGCTAGCTCCGGCCAT |
|  |  |  | Vrn-B1_I_ALG | CAACCTCCACGGTTTCAAAAAGTAG |
|  |  |  | Vrn-B1_I_C1 | ATATTTACTAAGCAGCGGTCATTCCGAT |
|  | Vrn-B1b | wMAS000037 | Vrn-B1_B_ALC | GCGCAAGCGGGAGCTACATC |
|  |  |  | Vrn-B1_B_ALG | TGCGCAAGCGGGAGCTACATG |
|  |  |  | Vrn-B1_B_C1 | GCCATGAACAACAAAGGGGGTGGT |
|  | Vrn-B1c | Vrn-B1_C | Vrn-B1_C _ALT | CCTAAACAGGGGCAGAACACTA |
|  |  |  | Vrn-B1_C _ALG | CCTAAACAGGGGCAGAACACTG |
|  |  |  | Vrn-B1_C _C | GACCCCAGGGCCTATGAATGTAATT |
|  | vrn-B1_intron1_A/C | TaVrn-B1_1752 | TaVrnB1_1752_AF2 | GGAATGACCGCTGCTTAGTAAATATA |
|  |  |  | TaVrnB1_1752_CF1 | GGAATGACCGCTGCTTAGTAAATATC |
|  |  |  | TaVrnB1_1752_R | GATTTAGCACCTCAACATACAGGTCT |
| Vrn-D1 | Vrn-D1a | wMAS000039 | Vrn-D1-D1a_A_ALC | ATCATTCGAATTGCTAGCTCCGC |
|  |  |  | Vrn-D1-D1a_A_ALG | ATCATTCGAATTGCTAGCTCCGG |
|  |  |  | Vrn-D1-D1a_A_C | GCCTGAACGCCTAGCCTGTGTA |
| Ppd-A1 | Ppd-A1a.1 | Ppd-A1prodel | Ppd-A1prodel_AL2 | GCGGCGAGCCGGTTAATCG |
|  |  |  | Ppd-A1prodel_AL1 | TTTCGGTGTTTGACTTCAGGCG |
|  |  |  | Ppd-A1prodel_C1 | GTGGCGTACTCCCTCCGTTTCTT |
| Ppd-B1 | Ppd-B1a Chinese Spring truncated copy | wMAS000027 | TaPpdBJ001tR | GACGTTATGAACGCTTGGCA |
|  |  |  | TaPpdBJ001iR | CCGTTTTCGCGGCCTT |
|  |  |  | TaPpdBJ001tF | GGGTTCGTCGGGAGCTGT |
|  | Ppd-B1a Sonora64 type intercopy | TaPpdBJ003 | TaPpdBJ003F | CGTGAAGAGCTAGCGATGAACA |
|  |  |  | TaPpdBJ003R | TGGGCACGTTAACACACCTTT |
| Ppd-D1 | Ppd-D1a Ciano67 promoter deletion | wMAS000024 | TaPpdDD001RI | CAAGGAAGTATGAGCAGCGGTT |
|  |  |  | TaPpdDD001RD | AAGAGGAAACATGTTGGGGTCC |
|  |  |  | TaPpdDD001FL | GCCTCCCACTACACTGGGC |
| Rht-B1 | Rht-B1b | wMAS000001 | RhtB1_SNP-AL1 | CCCATGGCCATCTCSAGCTG |
|  |  |  | RhtB1_SNP-AL2 | CCCATGGCCATCTCSAGCTA |
|  |  |  | RhtB1_SNP-C | TCGGGTACAAGGTGCGGGCG |
| Rht-D1 | Rht-D1b | wMAS000002 | RhtD1_AL1 | CATGGCCATCTCGAGCTRCTC |
|  |  |  | RhtD1_AL2 | CATGGCCATCTCGAGCTRCTA |
|  |  |  | RhtD1_C1 | CGGGTACAAGGTGCGCGCC |
