# Supplementary material for: Allelic Variation in Developmental Genes and Effects on Winter Wheat Heading Date in the U.S. Great Plains
Source: PLoS One. 2016 Apr 8;11(4):e0152852. doi: 10.1371/journal.pone.0152852 (PMC4825937; doi:10.1371/journal.pone.0152852)
Supplement: S3 Table — The environments are described in Table 1. The model terms were fit separately for each locus. The intercept (Int) describes the number of days from1 January to heading in each environment before the allelic effect is applied. The allelic effect at each locus is added to the Int value. Allelic effects were fit separately for each environment when there was significant genotype-by-environment (G×E) interaction at that locus in the combined analysis across all environments. Allelic effects from the combined analyses are reported for Ppd-A1 and vrn-B1 because significant G×E interaction was not observed at these loci. The grand mean heading date across all germplasm and environments was 131.8 ± 0.3 days. (DOCX) [file pone.0152852.s003.docx]

**S3 Table. Allelic effects (number of days) of photoperiod, reduced-height, and vernalization loci on heading date in each of nine environments.** The environments are described in Table 1. The model terms were fit separately for each locus. The intercept (Int) describes the number of days from1 January to heading in each environment before the allelic effect is applied. The allelic effect at each locus is added to the Int value. Allelic effects were fit separately for each environment when there was significant genotype-by-environment interaction (G×E) at that locus in the combined analysis across all environments. Allelic effects from the combined analyses are reported for *Ppd-A1* and *vrn-B1* because significant G×E was not observed at these loci. The grand mean heading date across all germplasm and environments was 131.8 ± 0.3 days.

|  | **CNV *vrn-A1*** |  |  | ***vrn-B1*** |  |
| --- | --- | --- | --- | --- | --- |
|  | **Int** | **CNV=1** | **CNV=2** | **Int** | **‘*Neuss*’ allele** |
| Ar13R | 109.51 | -4.67^***^ | -5.53^**^ | 106.81 | 2.22^**^ |
| Bu12R | 116.86 | -2.30^*^ | -1.48 ns^†^ | 114.43 | 2.22^**^ |
| Fo13 | 149.76 | -1.13^***^ | -0.45 ns | 147.43 | 2.22^**^ |
| Gr12F | 136.93 | -1.65^**^ | -1.29 ns | 134.56 | 2.22^**^ |
| Gr12P | 134.41 | -0.46^***^ | -0.68 ns | 132.17 | 2.22^**^ |
| Ha13R | 140.97 | -1.10^***^ | -0.74 ns | 138.66 | 2.22^**^ |
| Ma12 | 122.53 | -1.47^**^ | -3.54 ns | 120.14 | 2.22^**^ |
| It12R | 122.66 | -2.91 ns | -3.19 ns | 120.14 | 2.22^**^ |
| It13R | 145.80 | -1.27^**^ | -0.27 ns | 143.48 | 2.22^**^ |

**Supplemental Table S3.** Continued.

|  | ***Ppd-A1*** |  | ***Ppd-B1*** |  | ***Ppd-D1*** |  |
| --- | --- | --- | --- | --- | --- | --- |
|  | **Int** | ***‘b’* allele** | **Int** | ***‘b’* allele** | **Int** | ***‘b’* allele** |
| Ar13R | 107.02 | 1.97^***^ | 105.95 | 5.23^***^ | 105.33 | 5.17^***^ |
| Bu12R | 114.66 | 1.97^***^ | 114.23 | 4.04^***^ | 113.22 | 4.83^***^ |
| Fo13 | 147.70 | 1.97^***^ | 149.17 | 0.77^**^ | 149.00 | 0.91^**^ |
| Gr12F | 134.81 | 1.97^***^ | 135.29 | 2.50^***^ | 134.71 | 2.91^***^ |
| Gr12P | 132.41 | 1.97^***^ | 133.11 | 2.07^***^ | 132.76 | 2.27^***^ |
| Ha13R | 138.93 | 1.97^***^ | 140.14 | 1.23^***^ | 140.00 | 1.21^***^ |
| Ma12 | 120.37 | 1.97^***^ | 119.93 | 4.10^***^ | 119.24 | 4.40^***^ |
| It12R | 120.36 | 1.97^***^ | 119.50 | 4.81^***^ | 118.70 | 5.17^***^ |
| It13R | 143.73 | 1.97^***^ | 144.47 | 2.04^***^ | 144.44 | 1.74^***^ |

^*^, ^**^, ^***^ indicates significance at the 0.05, 0.01, and 0.001 probability levels, respectively.

**Supplemental Table S3.** Continued.

|  | ***Rht-B1*** |  |
| --- | --- | --- |
|  | **Int** | ***‘b’* allele** |
| Ar13R | 112.02 | 4.21^***^ |
| Bu12R | 119.25 | 3.67^***^ |
| Fo13 | 150.53 | 1.21^***^ |
| Gr12F | 137.91 | 1.59^**^ |
| Gr12P | 135.89 | 2.11 ^***^ |
| Ha13R | 141.66 | 1.13^**^ |
| Ma12 | 123.99 | 2.29^***^ |
| It12R | 124.93 | 3.61^***^ |
| It13R | 147.28 | 2.21^***^ |

^*^, ^**^, ^***^ indicates significance at the 0.05, 0.01, and 0.001 probability levels, respectively.
