# Supplementary material for: Allelic Variation in Developmental Genes and Effects on Winter Wheat Heading Date in the U.S. Great Plains
Source: PLoS One. 2016 Apr 8;11(4):e0152852. doi: 10.1371/journal.pone.0152852 (PMC4825937; doi:10.1371/journal.pone.0152852)
Supplement: S4 Table — Allelic effects and interaction of Ppd-D1b and Ppd-B1b on winter wheat heading date in each of nine environments, and proportion of variability (R2) in heading date explained by all terms in each model. The environments are described in Table 1. The model terms were fit separately for each environment. The intercept (Int) describes the number of days from 1 January to heading in each environment before the allelic effects are applied. The allelic effect (number of days) at each locus is added to the Int value. (DOCX) [file pone.0152852.s004.docx]

**S4 Table. Allelic effects (number of days) of photoperiod sensitive alleles *Ppd-D1b* and *Ppd-B1b.***  Allelic effects and interaction of *Ppd-D1b* and *Ppd-B1b* on winter wheat heading date in each of nine environments, and proportion of variability (*R^2^*) in heading date explained by all terms in each model. The environments are described in Table 1. The model terms were fit separately for each environment. The intercept (Int) describes the number of days from 1 January to heading in each environment before the allelic effects are applied. The allelic effect (number of days) at each locus is added to the Int value.

| **Env** | **Int** | ***Ppd-B1b*** | ***Ppd-D1b*** | ***PpdD1b*PpdB1b*** | ***R^2^*** |
| --- | --- | --- | --- | --- | --- |
| Ar13R | 105.45 | -0.20 ns^†^ | 0.65 ns | 8.59^***^ | 0.54 |
| Bu12R | 113.18 | -0.05 ns | 1.35^*^ | 6.65^***^ | 0.55 |
| Fo13 | 149.07 | -0.19 ns | 0.13 ns | 1.53^**^ | 0.12 |
| Gr12F | 134.68 | -0.05 ns | 0.79 ns | 4.13^***^ | 0.40 |
| Gr12P | 132.38 | 0.57 ns | 0.94 ns | 2.52^***^ | 0.26 |
| Ha13R | 139.79 | 0.25 ns | 0.45 ns | 1.61 ns | 0.30 |
| Ma12 | 119.18 | 0.09 ns | 0.97 ns | 6.43^***^ | 0.65 |
| It12R | 118.77 | -0.20 ns | 0.94 ns | 7.99^***^ | 0.61 |
| It13R | 144.60 | -0.31 ns | -0.18 ns | 3.62^***^ | 0.27 |

^*^, ^**^, ^***^ indicates significance at the 0.05, 0.01, and 0.001 probability levels, respectively.

^†^ ns indicates non-significance at the 0.05 probability level.
